# Supplementary material for: Genetic ancestry, skin pigmentation, and the risk of cutaneous squamous cell carcinoma in Hispanic/Latino and non-Hispanic white populations
Source: Commun Biol. 2020 Dec 14;3:765. doi: 10.1038/s42003-020-01461-8 (PMC7736583; doi:10.1038/s42003-020-01461-8)
Supplement: Supplementary file 1 — Supplementary Information [file 42003_2020_1461_MOESM1_ESM.pdf]

Supplementary Information for 'Genetic ancestry and pigmentation specifically affect risk of cutaneous squamous cell carcinoma among Hispanic/Latino and non-Hispanic white populations'

Eric Jorgenson[†], Hélène Choquet[†], Jie Yin, Thomas J. Hoffmann, Yambazi Banda, Mark N. Kvale, Neil Risch,

Catherine Schaefer, and Maryam M. Asgari

[†]These authors contributed equally

Supplementary Figure 1: Ancestry contour figures showing the probability of each of the skin pigmentation traits versus the first two principal components of genetic ancestry in GERA non-Hispanic whites. a. very pale or pale skin color; b. intermediate skin color; c. dark or dark to black skin color. The probability of each type of skin pigmentation is indicated on a color scale, with warmer colors representing high probabilities of that skin type. Axes reflect the first two principal components of ancestry. Nationality subgroup labels were derived from the Human Genome Diversity Project populations.

a.

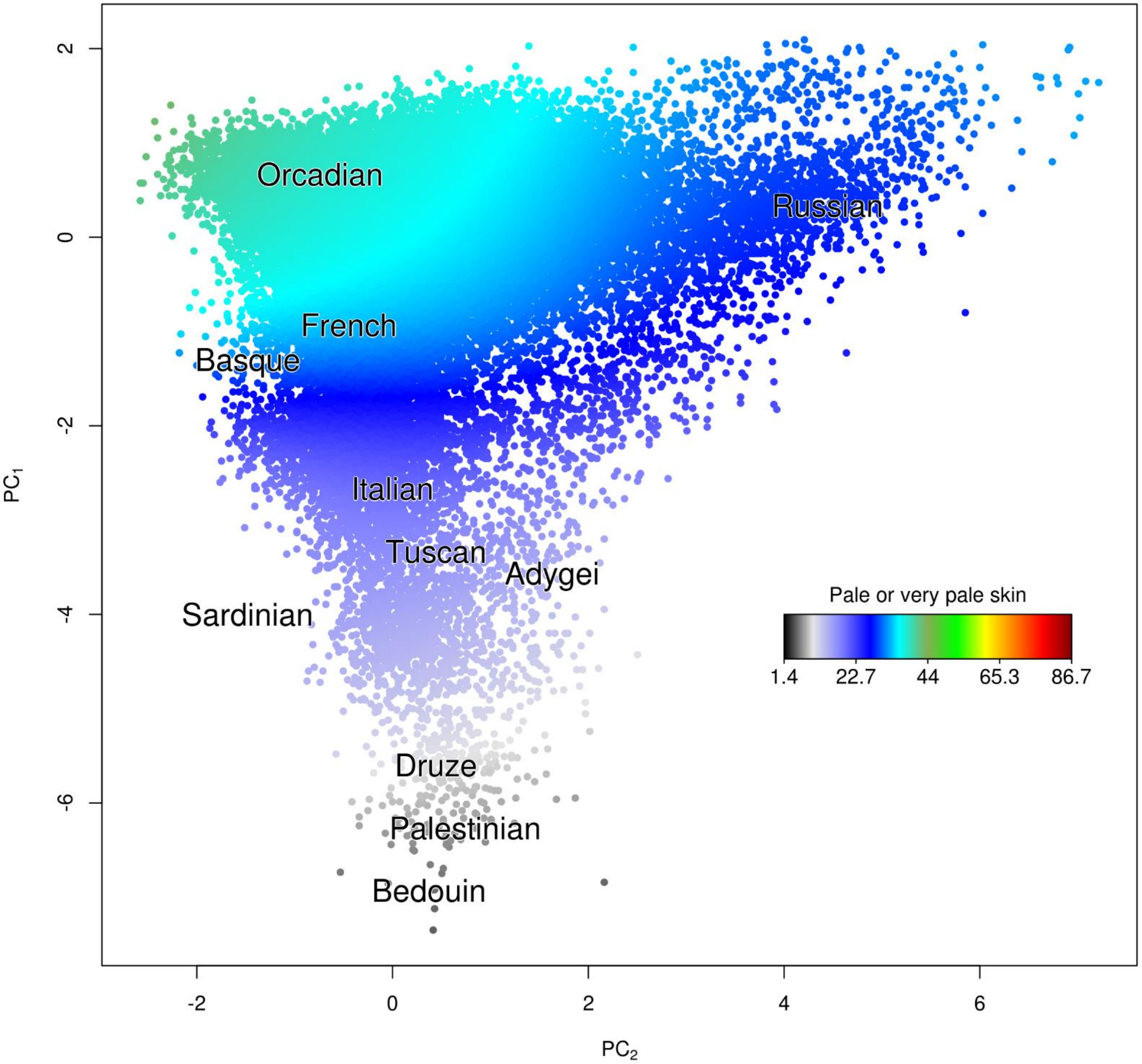

b.

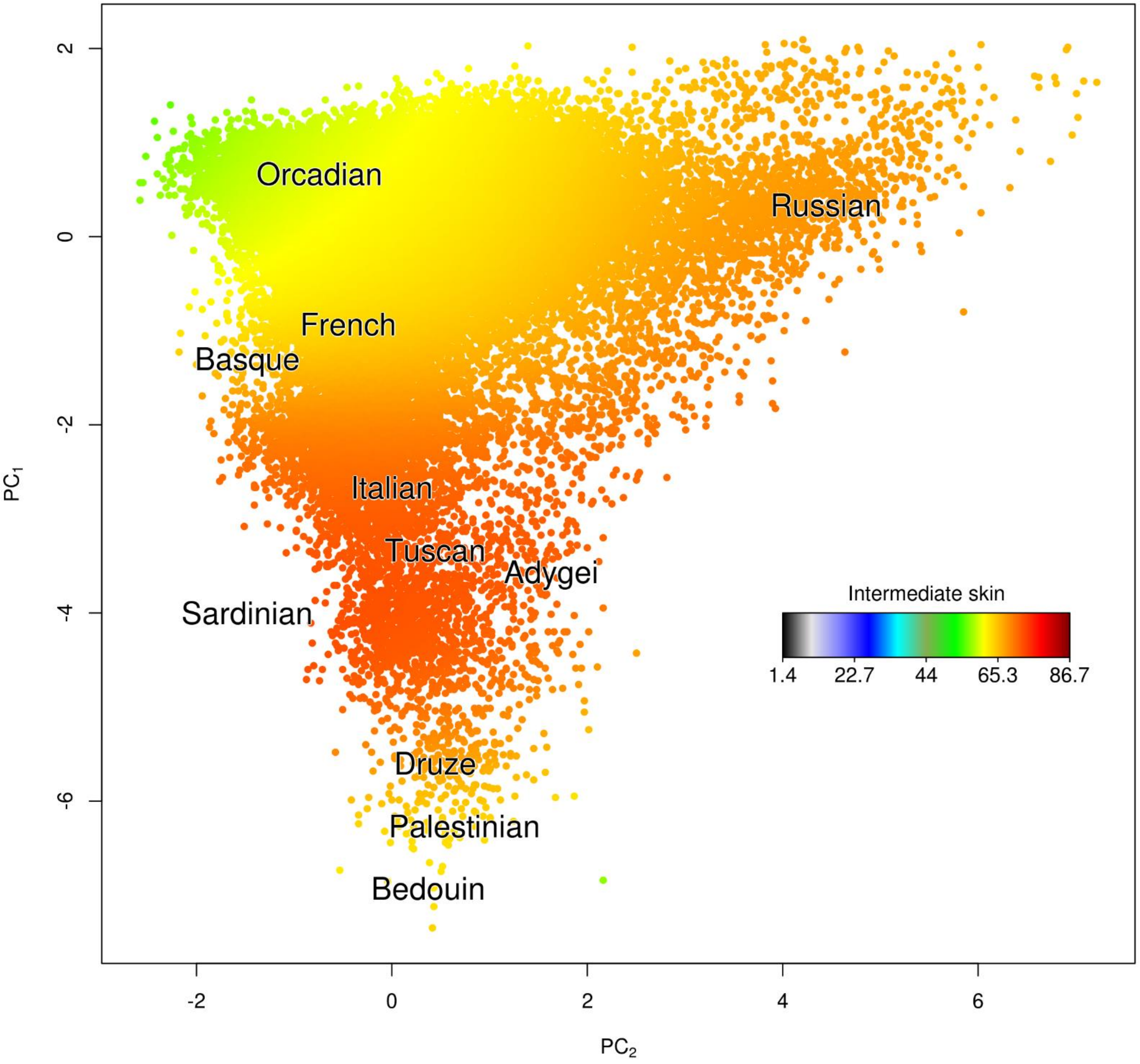

Orcadian
Russian
French
Basque
Italian
Tuscan
Adygei
Sardinian
Druze
Palestinian
Bedouin
Intermediate skin
1.4
22.7
44
65.3
86.7
PC1
PC2
2
0
-2
-4
-6
-2
0
2
4
6

c.

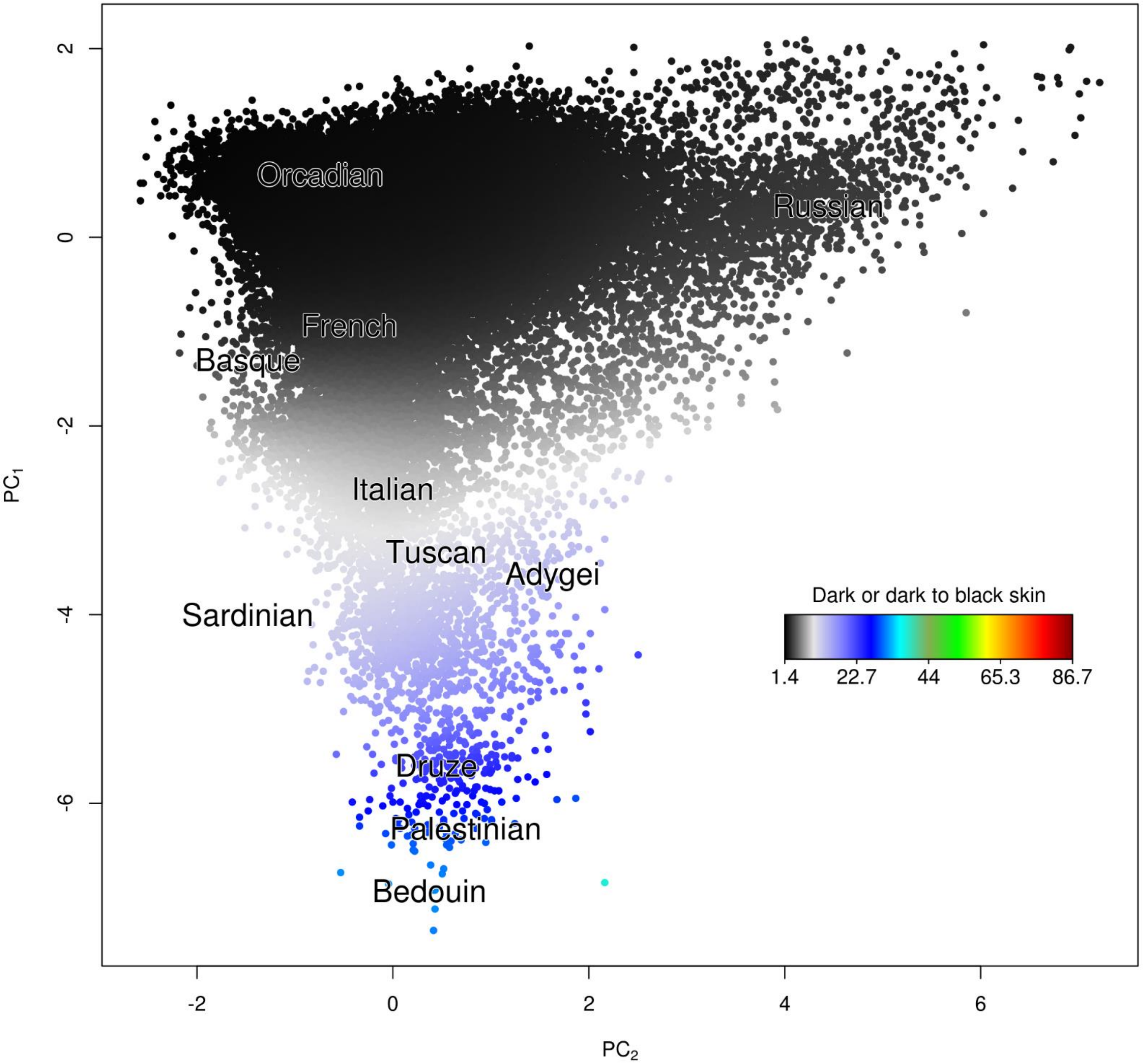

Orcadian
Russian
French
Basque
Italian
Tuscan
Adygei
Sardinian
Druze
Palestinian
Bedouin
Dark or dark to black skin
1.4
22.7
44
65.3
86.7
2
0
-2
-4
-6
-2
0
2
4
6
PC1
PC2

Supplementary Figure 2: Ancestry contour figures showing the probability of each of the skin pigmentation traits versus the first two principal components of genetic ancestry in GERA Hispanic/Latinos. a. very pale or pale skin color; b. intermediate skin color; c. dark or dark to black skin color. The probability of each type of skin pigmentation is indicated on a color scale, with warmer colors representing high probabilities of that skin type. Axes reflect the first two principal components of ancestry. Nationality subgroup labels were derived from the Human Genome Diversity Project populations.

a.

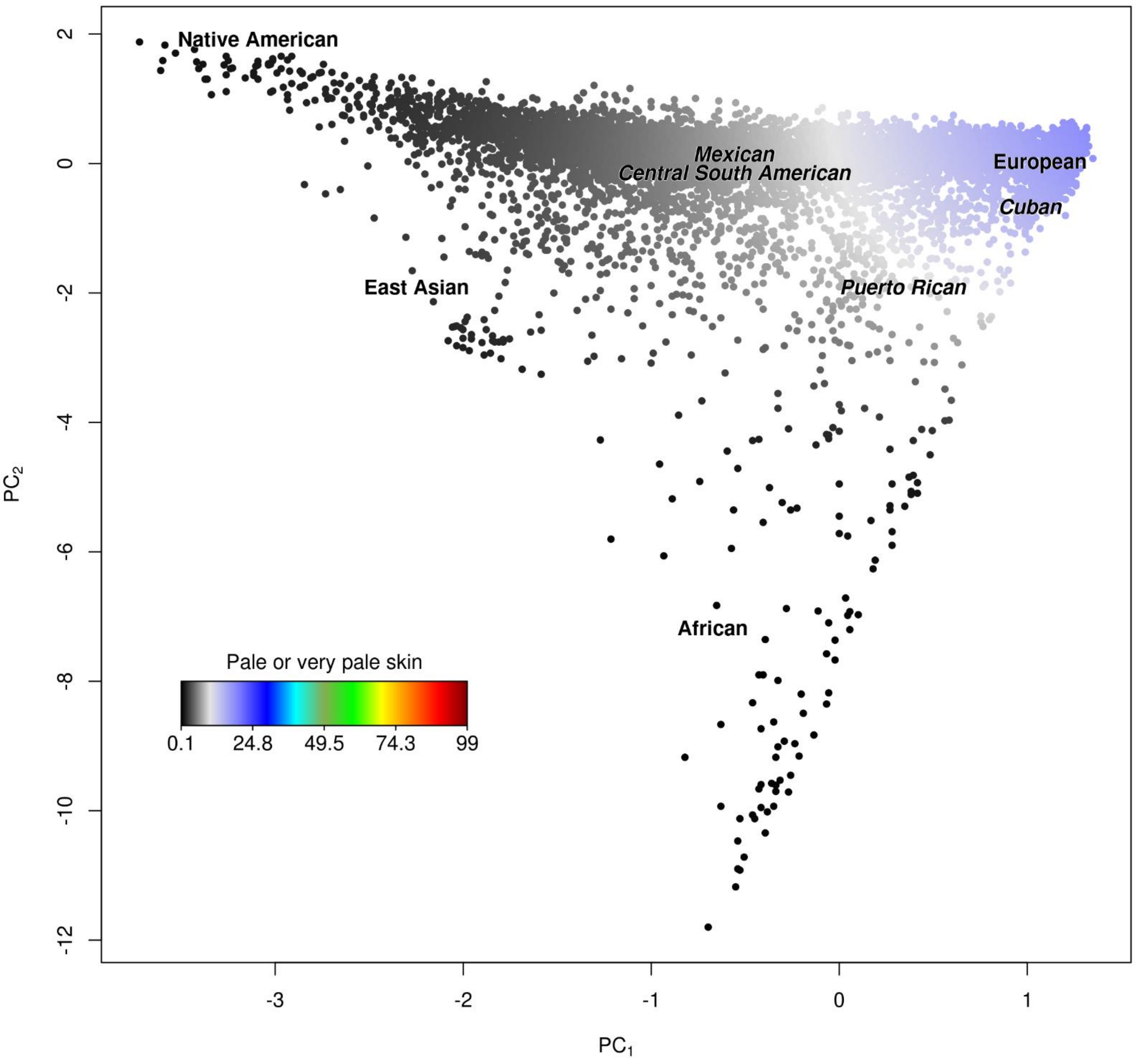

b.

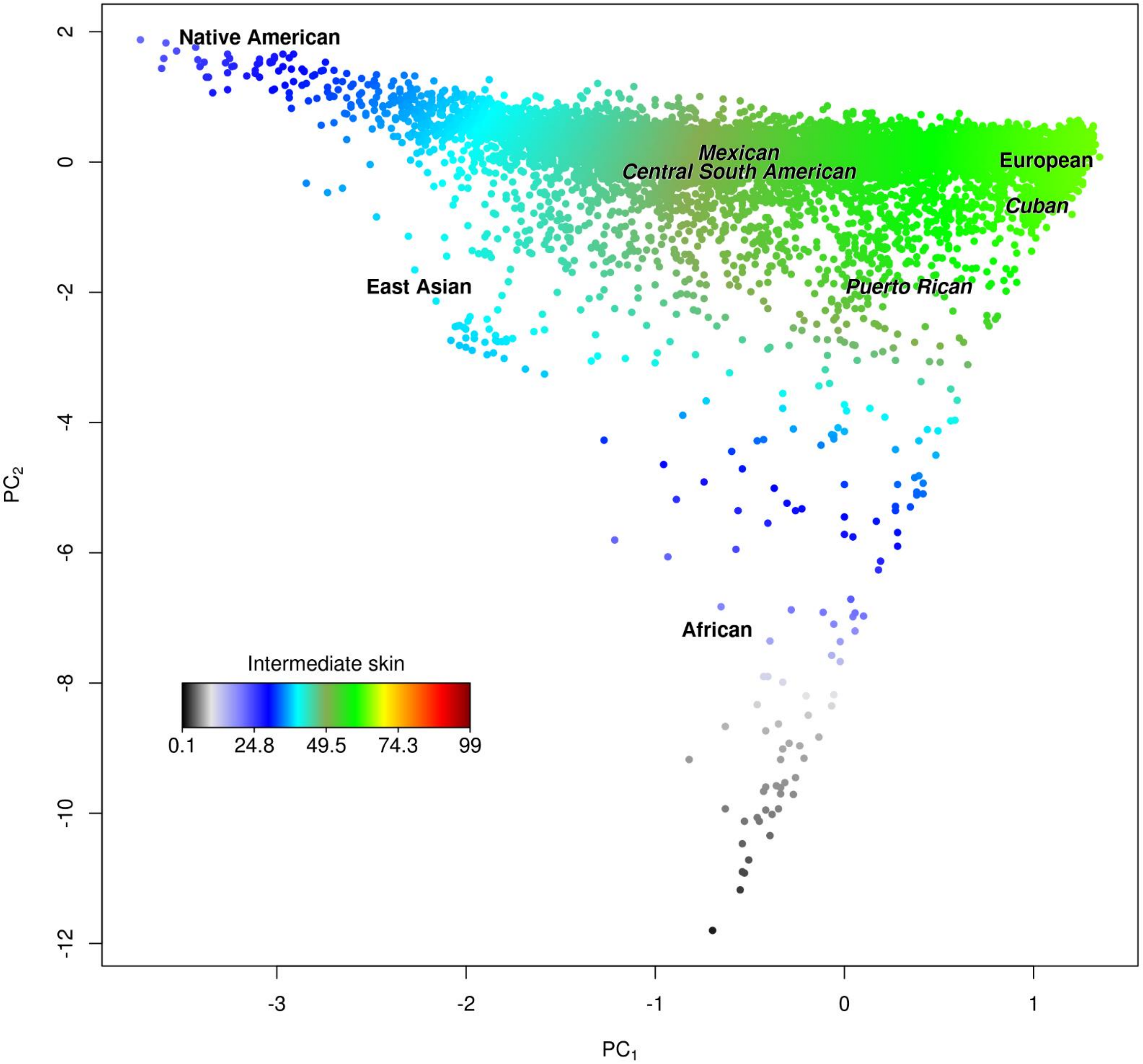

Native American
Mexican
Central South American
European
Cuban
East Asian
Puerto Rican
African
Intermediate skin
0.1
24.8
49.5
74.3
99
2
0
-2
-4
-6
-8
-10
-12
-3
-2
-1
0
1
PC_1
PC_2

c.

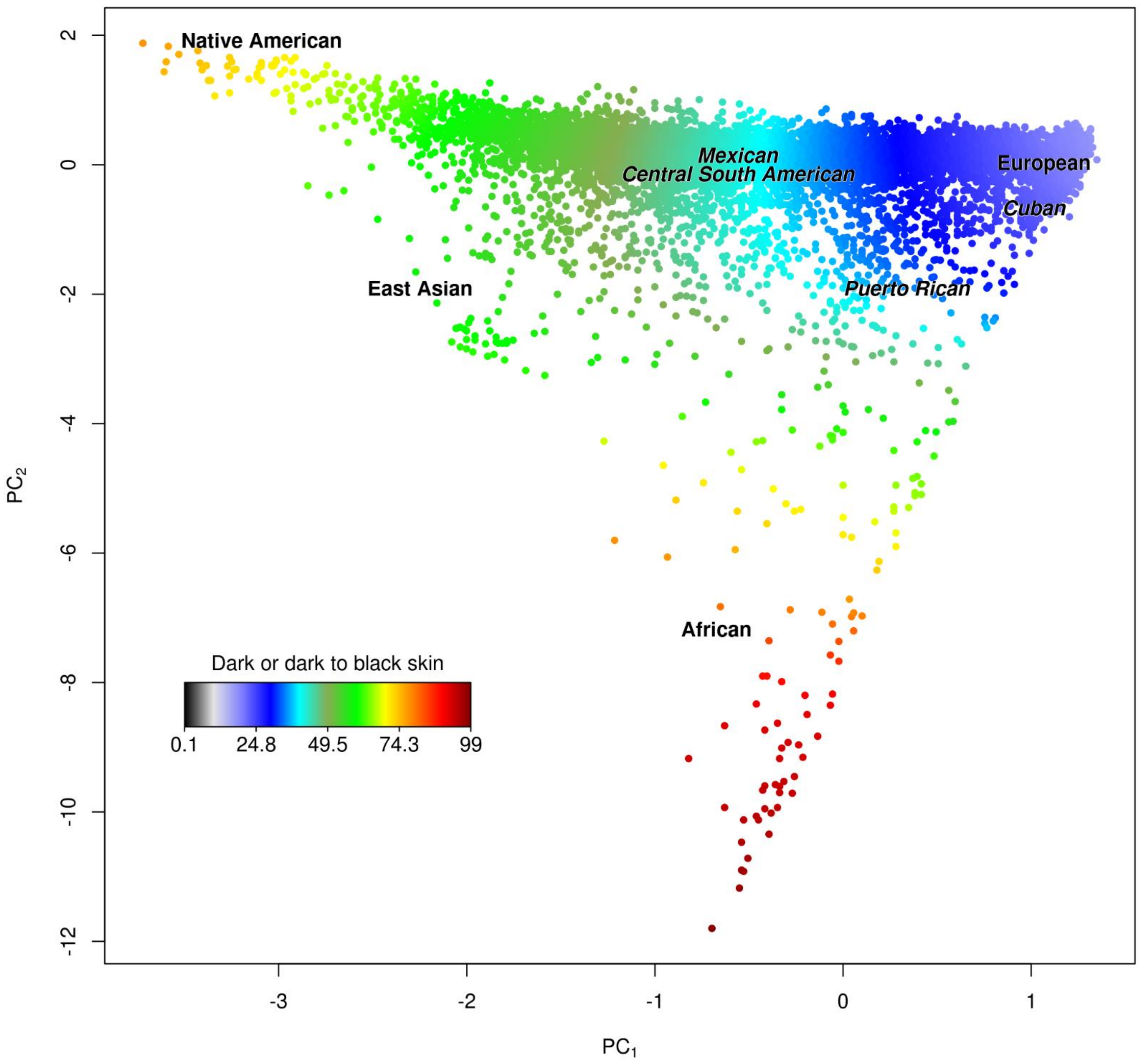

Native American
Mexican
Central South American
European
Cuban
East Asian
Puerto Rican
African
Dark or dark to black skin
0.1
24.8
49.5
74.3
99
2
0
-2
-4
-6
-8
-10
-12
-3
-2
-1
0
1
PC_2
PC_1

Supplementary Figure 3: Ancestry contour figure showing cSCC prevalence by genetic ancestry, stratified by tumor location (sun exposed or sun protected). a. cSCC risk at sun protected sites within GERA non-Hispanic whites; b. cSCC risk at sun exposed sites within GERA non-Hispanic whites; c. cSCC risk at sun protected sites within GERA Hispanic/Latinos; d. cSCC risk at sun exposed sites within GERA Hispanic/Latinos. cSCC prevalence is indicated on a color scale, with warmer colors indicating higher prevalence. Axes reflect the first two principal components of ancestry. Nationality subgroup labels were derived from the Human Genome Diversity Project populations.

a.

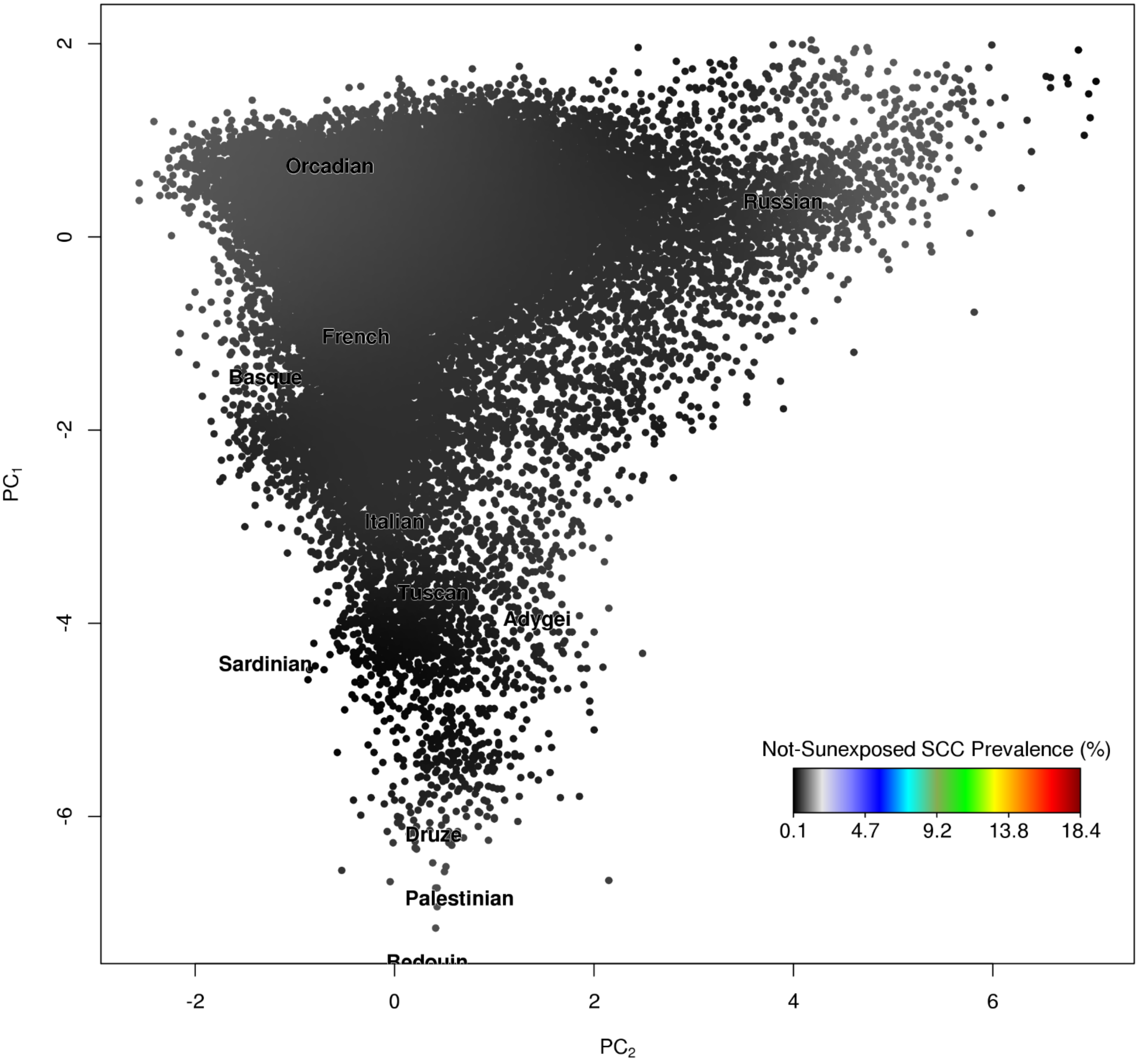

b.

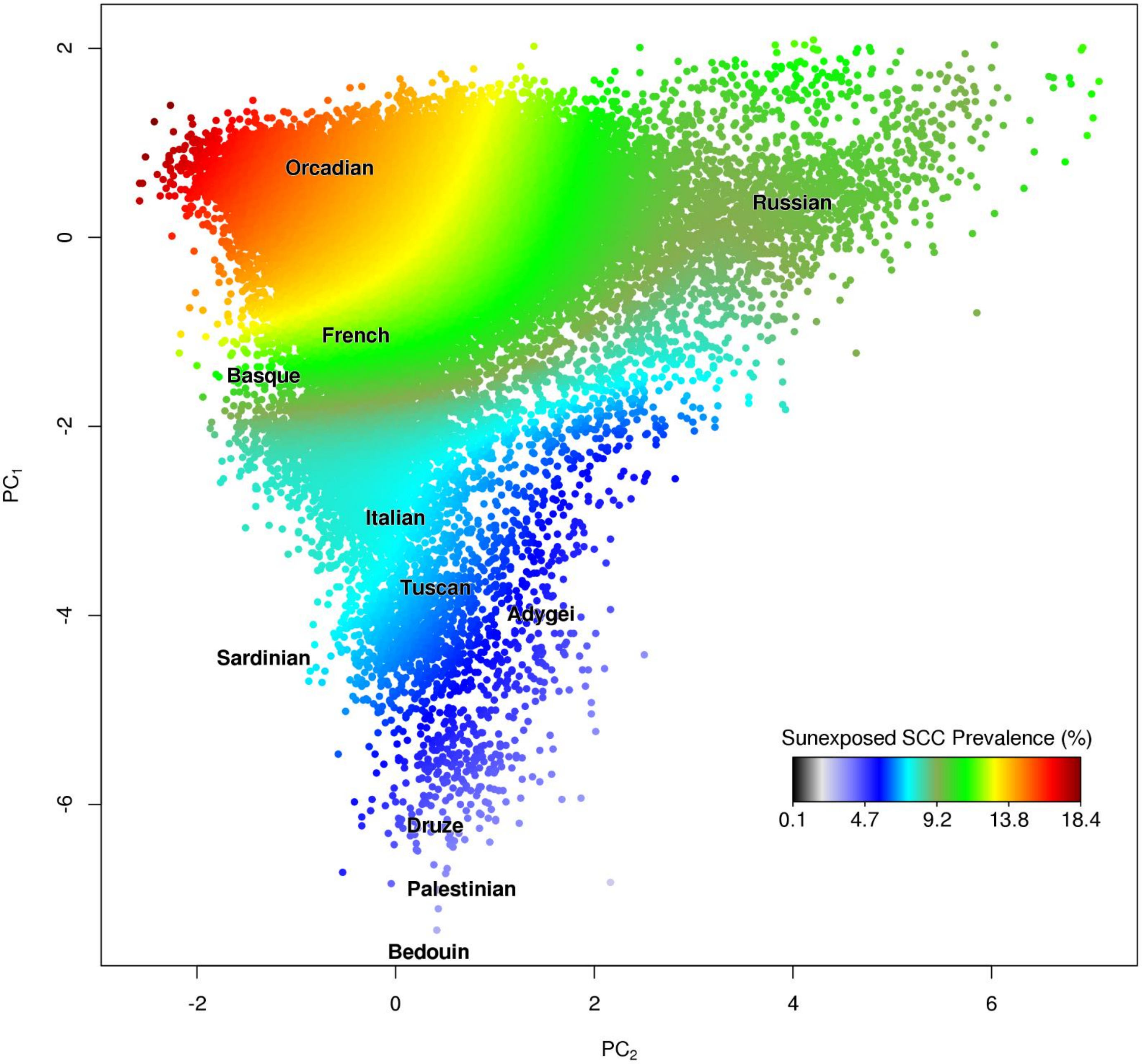

Orcadian
Russian
French
Basque
Italian
Tuscan
Adygei
Sardinian
Druze
Palestinian
Bedouin
Sunexposed SCC Prevalence (%)
0.1
4.7
9.2
13.8
18.4
PC1
PC2
2
0
-2
-4
-6
-2
0
2
4
6

c.

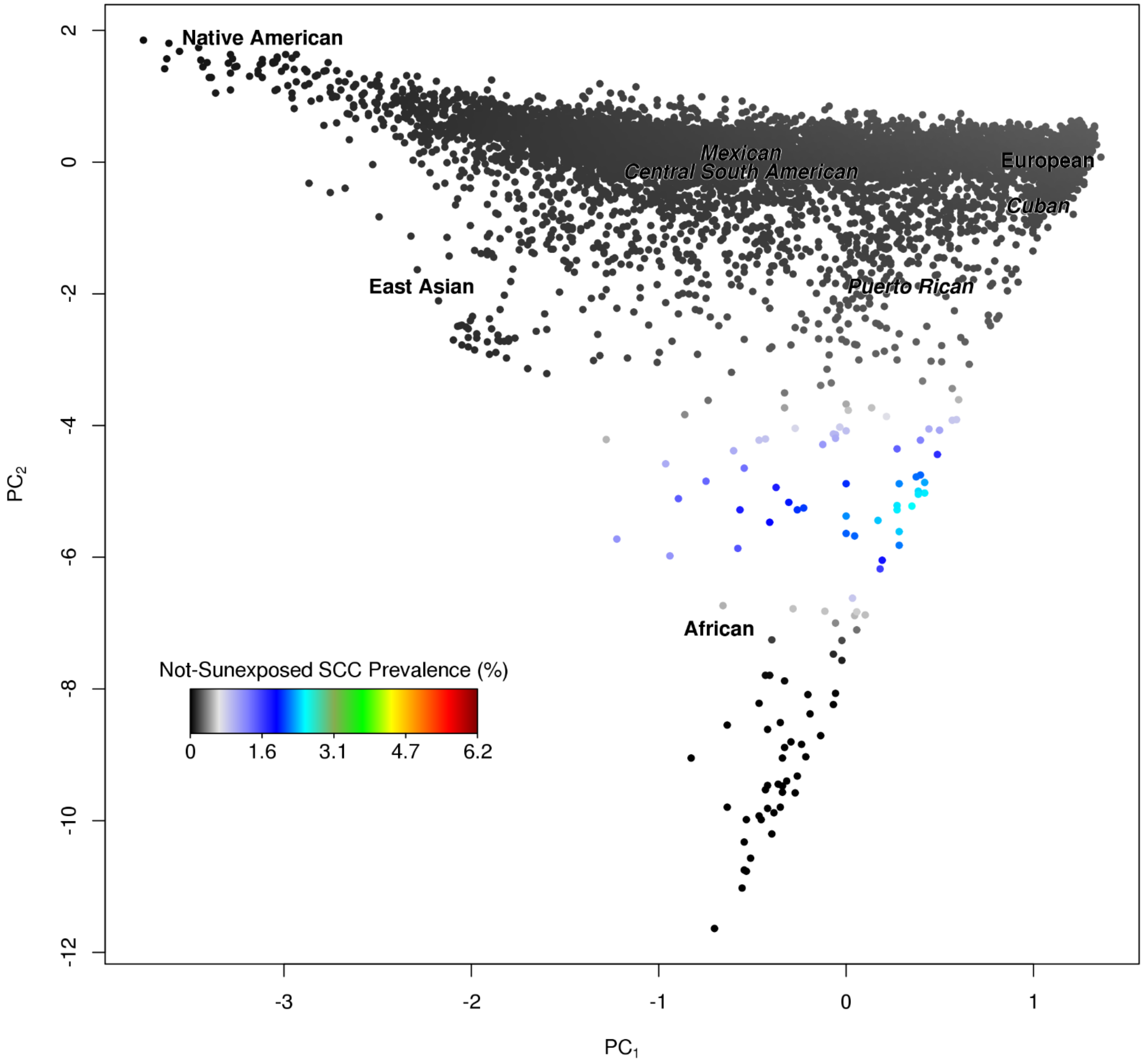

Native American
Mexican
Central South American
European
Cuban
East Asian
Puerto Rican
African
Not-Sunexposed SCC Prevalence (%)
0
1.6
3.1
4.7
6.2
PC2
PC1
2
0
-2
-4
-6
-8
-10
-12
-3
-2
-1
0
1

d.

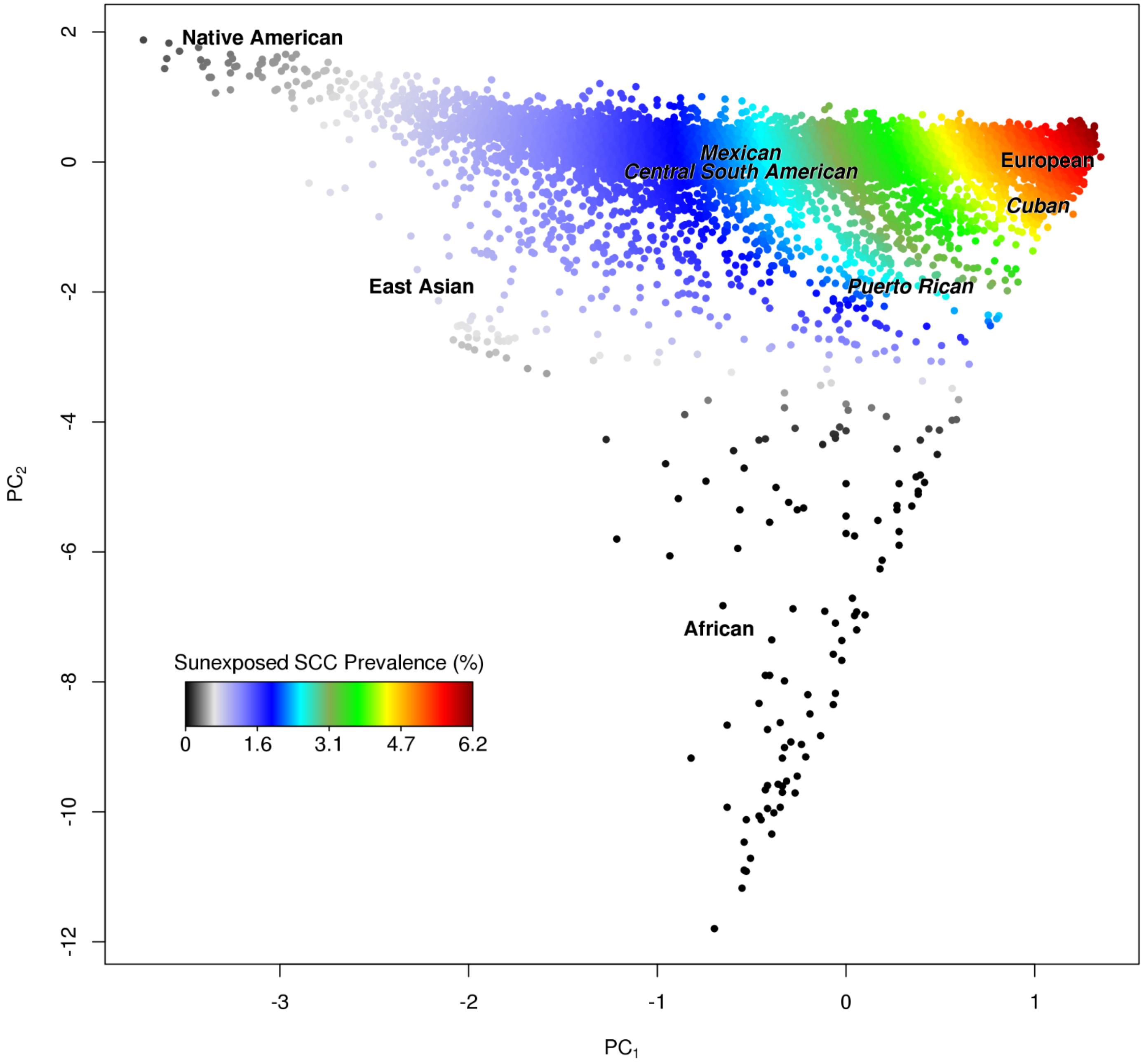

Native American
Mexican
Central South American
European
Cuban
East Asian
Puerto Rican
African
Sunexposed SCC Prevalence (%)
0
1.6
3.1
4.7
6.2
PC2
PC1
2
0
-2
-4
-6
-8
-10
-12
-3
-2
-1
0
1

Supplementary Figure 4: Ancestry contour figure showing non-cutaneous SCC prevalence by genetic ancestry in GERA. a. non-Hispanic whites; b. Hispanic/Latinos. ncSCC prevalence is indicated on a color scale, with warmer colors indicating higher prevalence. Axes reflect the first two principal components of ancestry. Nationality subgroup labels were derived from the Human Genome Diversity Project populations.

a.

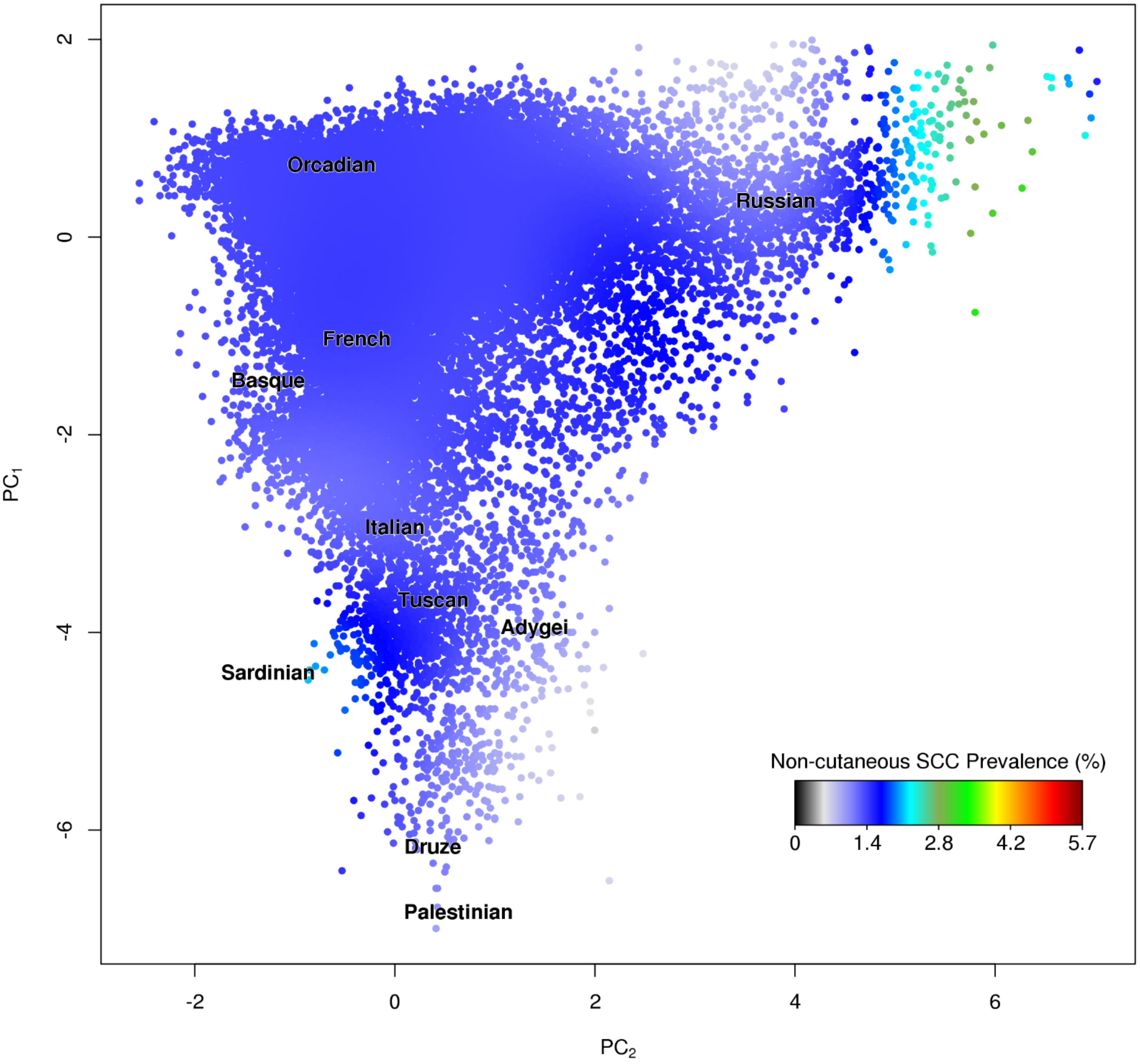

b.

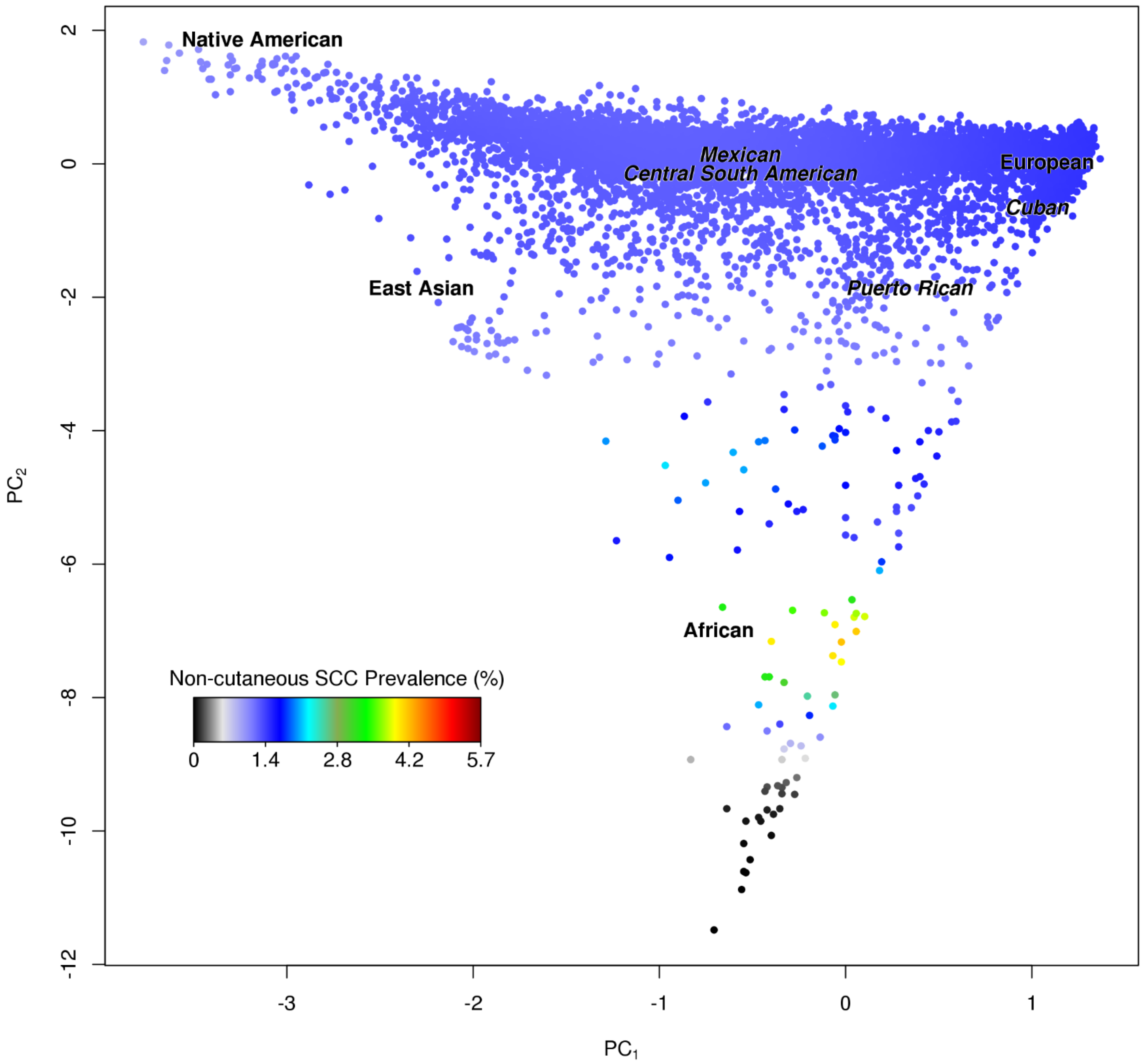

Native American
Mexican
Central South American
European
Cuban
East Asian
Puerto Rican
African
Non-cutaneous SCC Prevalence (%)
0
1.4
2.8
4.2
5.7
2
0
-2
-4
-6
-8
-10
-12
-3
-2
-1
0
1
PC_1
PC_2

Supplementary Table 1. Multivariate logistic regression models of cSCC in GERA Hispanic/Latinos

	Model 1		Model 2		Model 3		Model 4	
	**β (SE)**	***P***	**β (SE)**	***P***	**β (SE)**	***P***	**β (SE)**	***P***
**Age**	0.075 (0.0056)	$1.78x10^{-41}$	0.078 (0.006)	$1.27x10^{-41}$	0.078 (0.006)	$7.08x10^{-42}$	0.066 (0.0062)	$1.30x10^{-26}$
**Sex**	-0.55 (0.13)	$2.06\ x10^{-5}$	-0.54 (0.13)	$3.25x10^{-5}$	-0.54 (0.13)	$3.33x10^{-5}$	-0.42 (0.14)	$2.11x10^{-3}$
**PC1**	122.48 (8.95)	$1.27x10^{-42}$	67.31 (11.19)	$1.77x10^{-9}$	56.54 (11.49)	$8.54x10^{-7}$	41.30 (11.98)	$5.67x10^{-4}$
**PC2**	43.52 (15.97)	0.0064	28.43 (15.80)	0.072	28.44 (15.63)	0.069	18.67 (15.17)	0.22
**PC3**	12.64 (12.27)	0.30	10.17 (11.77)	0.39	11.38 (11.96)	0.34	9.47 (12.00)	0.43
**PC4**	10.95 (6.68)	0.10	1.15 (6.72)	0.86	1.90 (6.68)	0.78	3.01 (6.74)	0.66
**PC5**	22.50 (8.30)	0.0067	20.56 (8.28)	0.013	20.91 (8.31)	0.01	16.01 (8.53)	0.06
**PC6**	-12.23 (7.64)	0.11	-10.58 (7.63)	0.17	-9.76 (7.63)	0.20	-7.54 (7.77)	0.33
**Intermediate skin**	-	-	-2.23 (0.31)	$1.54x10^{-12}$	-1.31 (0.37)	$4.54x10^{-4}$	-0.94 (0.39)	0.016
**Dark skin**	-	-	-3.22 (0.39)	$1.09x10^{-16}$	-2.14 (0.46)	$2.93x10^{-6}$	-1.62 (0.47)	$5.54x10^{-4}$
**GRS**	-	-	-	-	0.13 (0.03)	$1.26x10^{-5}$	0.12 (0.03)	$6.84x10^{-5}$
**AK**	-	-	-	-	-	-	1.64 (0.14)	$5.12x10^{-30}$

Abbreviations: PC, principal component; β, beta; SE, standard error; GRS, genetic risk score (based on 14 SNPs previously reported to be associated with cSCC risk), AK, actinic keratosis.

Note: The first two principal components (PC1 and PC2), which represent geographic origin, are highlighted in yellow. Model 1: Ancestry; Model 2: Model 1 and HIrisPlex-S skin pigmentation prediction; Model 3: Model 2 and Genetic Risk Score; Model 4: Model 3 and AK.

In models 2, 3 and 4, the HIrisPlex-S prediction for the combination of very pale skin with pale skin served as the reference group.

Supplementary Table 2. Multivariate logistic regression models of cSCC in GERA non-Hispanic Whites

	Model 1		Model 2		Model 3		Model 4	
	**β (SE)**	***P***	**β (SE)**	***P***	**β (SE)**	***P***	**β (SE)**	***P***
**Age**	0.067 (0.0010)	$<2.23x10^{-308}$	0.067 (0.0010)	$<2.23x10^{-308}$	0.068 (0.0010)	$<2.23x10^{-308}$	0.052 (0.0011)	$<2.23x10^{-308}$
**Sex**	-0.56 (0.022)	$1.75x10^{-145}$	-0.56 (0.022)	$7.63x10^{-145}$	-0.56 (0.022)	$1.91x10^{-145}$	-0.43 (0.023)	$1.04x10^{-78}$
**PC1**	30.12 (1.76)	$2.38x10^{-65}$	16.53 (1.84)	$2.42x10^{-19}$	17.20 (1.84)	$1.07x10^{-20}$	11.49 (1.90)	$1.52x10^{-9}$
**PC2**	-25.23 (1.71)	$2.28x10^{-49}$	-19.36 (1.73)	$5.33x10^{-29}$	-18.33 (1.74)	$4.41x10^{-26}$	-12.99 (1.80)	$5.37x10^{-13}$
**PC3**	-13.74 (1.75)	$4.27 x 10^{-15}$	-10.30 (1.76)	$5.30x10^{-9}$	-8.52 (1.77)	$1.50x10^{-6}$	-6.75 (1.84)	$2.38x10^{-4}$
**PC4**	-0.02 (1.87)	0.99	-0.70 (1.88)	0.71	-0.78 (1.89)	0.68	-0.84 (1.95)	0.66
**PC5**	4.23 (2.24)	0.059	4.24 (2.26)	0.060	4.12 (2.26)	0.068	3.23 (2.33)	0.17
**PC6**	1.24 (1.98)	0.53	1.02 (2.01)	0.61	1.50 (2.02)	0.46	2.02 (2.09)	0.33
**PC7**	3.45 (2.0)	0.085	2.86 (2.01)	0.15	2.83 (2.00)	0.16	2.46 (2.07)	0.23
**PC8**	-2.67 (2.0)	0.18	-2.35 (2.01)	0.24	-2.51 (2.02)	0.21	-1.96 (2.08)	0.35
**PC9**	-1.98 (1.99)	0.32	-2.21 (2.01)	0.27	-2.28 (2.02)	0.26	-3.23 (2.09)	0.12
**PC10**	2.33 (2.02)	0.25	-0.05 (2.05)	0.98	0.18 (2.05)	0.93	-0.97 (2.13)	0.65
**ASHK**	0.82 (0.083)	$2.12 x 10^{-23}$	0.52 (0.084)	$7.46x10^{-10}$	0.49 (0.08)	$7.71x10^{-9}$	0.24 (0.09)	$6.36x10^{-3}$
**Intermediate skin**	-	-	-1.32 (0.05)	$6.97x10^{-150}$	-0.68 (0.06)	$5.94x10^{-26}$	-0.45 (0.07)	$1.26x10^{-11}$
**Dark skin**	-	-	-2.24 (0.17)	$3.47x10^{-41}$	-1.46 (0.17)	$4.44x10^{-17}$	-0.96 (0.18)	$4.84x10^{-8}$
**GRS**	-	-	-	-	0.088 (0.0058)	$8.50x10^{-54}$	0.067 (0.006)	$1.14x10^{-29}$
**AK**	-	-	-	-	-	-	1.39 (0.024)	$<2.23x10^{-308}$

Abbreviations: PC, principal component; β, beta; SE, standard error; ASHK, Ashkenazi ancestry proportion; GRS, genetic risk score (based on 14 SNPs previously reported to be associated with cSCC risk), AK, actinic keratosis.

Note: The first two principal components (PC1 and PC2), which represent geographic origin, are highlighted in yellow. Model 1: Ancestry; Model 2: Model 1 and HIrisPlex-S skin pigmentation prediction; Model 3: Model 2 and Genetic Risk Score; Model 4: Model 3 and AK.

In models 2, 3 and 4, the HIrisPlex-S prediction for the combination of very pale skin with pale skin served as the reference group.

Supplementary Table 3. SNPs associated with human skin pigmentation variation in the HIrisPlex-S system

SNP	GENE	Chr	Position	Pigment Traits
rs16891982	*SLC45A2*	5	33951693	Eye, hair and skin color
rs28777	*SLC45A2*	5	33958959	Hair and skin color
rs12203592	*IRF4*	6	396321	Eye, hair and skin color
rs683	*TYRP1*	9	12709305	Hair and skin color
rs10756819	*BNC2*	9	16858084	Skin color
rs1042602	*TYR*	11	88911696	Hair and skin color
rs1393350	*TYR*	11	89011046	Eye and skin color
rs1126809	*TYR*	11	89017961	Skin color
rs12821256	*KITLG*	12	89328335	Hair and skin color
rs12896399	*LOC105370627*	14	92773663	Eye and skin color
rs2402130	*SLC24A4*	14	92801203	Hair and skin color
rs17128291	*SLC24A4*	14	92882826	Skin color
rs1545397	*OCA2*	15	28187772	Skin color
rs1800414	*OCA2*	15	28197037	Skin color
rs1800407	*OCA2*	15	28230318	Eye, hair and skin color
rs12441727	*OCA2*	15	28271775	Skin color
rs1470608	*OCA2*	15	28288121	Skin color
rs1129038	*HERC2*	15	28356859	Skin color
rs12913832	*HERC2*	15	28365618	Eye, hair and skin color
rs2238289	*HERC2*	15	28453215	Skin color
rs6497292	*HERC2*	15	28496195	Skin color
rs1667394	*HERC2*	15	28530182	Skin color
rs1426654	*SLC24A5*	15	48426484	Skin color
rs3114908	*ANKRD11*	16	89383725	Skin color
rs3212355	*MC1R*	16	89984378	Skin color
rs1805006	*MC1R*	16	89985918	Hair and skin color
rs2228479	*MC1R*	16	89985940	Hair and skin color
rs11547464	*MC1R*	16	89986091	Hair and skin color
rs1805007	*MC1R*	16	89986117	Hair and skin color
rs1110400	*MC1R*	16	89986130	Hair and skin color
rs1805008	*MC1R*	16	89986144	Hair and skin color
rs885479	*MC1R*	16	89986154	Hair and skin color
rs8051733	*DEF8*	16	90024206	Skin color
rs6059655	*RALY*	20	32665748	Skin color
rs6119471	*ASIP*	20	32785212	Skin color
rs2378249	*PIGU*	20	33218090	Hair and skin color

Table modified from https://hirisplex.erasmusmc.nl/;

Supplementary Table 4. List of the 16 cSCC-associated SNPs in previous cSCC GWAS

SNP	Chr Region	Locus	Ref
rs192481803	2p22	-	Chahal et al. 2016
rs62246017	3p13	*FOXP1*	Asgari et al. 2016
rs6791479	3q28	*TPRG1/TP63*	Asgari et al. 2016
rs35407	5p13	*SLC45A2*	Chahal et al. 2016
rs12203592	6p25	*IRF4*	Chahal et al. 2016
rs4455710	6p21	*HLA-DQA1*	Asgari et al. 2016
rs117132860	7p21	*AHR*	Chahal et al. 2016
rs57994353	9q34	*SEC16A*	Chahal et al. 2016
rs10810657	9p22	*BNC2/CNTLN*	Asgari et al. 2016
rs1126809	11q14	*TYR*	Chahal et al. 2016
rs74899442	11q23	*CADM1*	Chahal et al. 2016
rs1800407	15q13.1	*OCA2*	Chahal et al. 2016
rs12916300	15q13.1	*OCA2/HERC2*	Asgari et al. 2016
rs1805007	16q24.3	*MC1R*	Chahal et al. 2016
rs4268748	16q24.3	*DEF8*	Asgari et al. 2016
rs6059655	20q11	*RALY*	Chahal et al. 2016

Supplementary Table 5. Correlation between skin pigmentary-SNPs from HirisPlex-S and previously reported cSCC-SNPs located within the same locus

**SNP[a]**	**Chr region**	**Locus**	**SNP[b]**	***R^2***	**D'**	**Distance (kb) between SNP[a] and SNP[b]**
rs16891982	5p13.2	*SLC45A2*	rs35407	0.6799	1	5
rs28777	5p13.2	*SLC45A2*	rs35407	0.9763	1	12
rs10756819	9p22.2	*BNC2*	rs10810657	0.531	0.8801	27
rs1393350	11q14.3	*TYR*	rs1126809	0.9532	1	7
rs1129038	15q13.1	*HERC2*	rs12916300	0.8316	0.9545	54
			rs1800407	0.0597	0.6478	127
rs12913832	15q13.1	*HERC2*	rs12916300	0.8433	0.9591	45
			rs1800407	0.0563	0.6277	135
rs1667394	15q13.1	*HERC2*	rs12916300	0.5914	0.9813	120
			rs1800407	0.0967	0.6173	300
rs6497292	15q13.1	*HERC2*	rs12916300	0.1834	0.9833	86
			rs1800407	0.0002	0.0163	266
rs8051733	16q24.3	*DEF8*	rs4268748	0.7296	0.9699	2
			rs1805007	0.1876	0.9617	38
rs1805008	16q24.3	*MC1R*	rs4268748	0.1625	0.8538	40
			rs1805007	0.0051	1	0.027
rs3114908	16q24.3	*ANKRD11*	rs4268748	0.0758	0.3407	643
			rs1805007	0.0831	0.6976	602
rs2378249	20q11.22	*PIGU*	rs6059655	0.277	0.9191	552

[a]index SNP associated with skin pigmentary traits from HirisPlex-S; [b]index SNP associated cSCC risk in previous studies; linkage disequilibrium (LD) metrics (R^2 and D') have all been calculated in European-ancestry populations using a web-based bioinformatic tool (https://analysistools.nci.nih.gov/LDlink/)[25]

Supplementary Table 6. Association of genetic ancestry with each skin pigmentation- and cSCC-associated SNP.

	PC1			PC2			Trait*
SNP	Beta	SE	P	Beta	SE	P	
rs16891982	-0.00725	0.000128	<2.23 x 10^{-308}	0.000942	0.000086	8.00 x 10^{-28}	Skin pigment
rs28777	-0.00686	0.000143	<2.23 x 10^{-308}	0.0009	0.000096	4.77 x 10^{-21}	Skin pigment
rs12203592	-0.00072	0.000073	2.70 x 10^{-23}	0.001742	0.000047	7.32 x 10^{-294}	Both
rs683	-0.00169	0.000057	1.10 x 10^{-190}	0.000172	0.000038	5.51 x 10^{-6}	Skin pigment
rs10756819	-0.00024	0.000060	4.92 x 10^{-5}	0.000451	0.000039	9.15 x 10^{-31}	Skin pigment
rs1042602	0.001528	0.000057	2.55 x 10^{-160}	0.000231	0.000037	6.12 x 10^{-10}	Skin pigment
rs1393350	-0.00097	0.000063	4.32 x 10^{-53}	0.000543	0.000041	4.05 x 10^{-39}	Skin pigment
rs1126809	-0.00096	0.000062	2.26 x 10^{-54}	0.000543	0.000041	2.15 x 10^{-40}	Both
rs12821256	-0.00319	0.000092	4.01 x 10^{-262}	0.000205	0.000061	0.00077	Skin pigment
rs12896399	-0.00161	0.000055	3.50 x 10^{-186}	0.000028	0.000037	0.45	Skin pigment
rs2402130	-0.00038	0.000069	4.95 x 10^{-8}	-0.00044	0.000046	5.84 x 10^{-22}	Skin pigment
rs17128291	-0.00036	0.000080	6.57 x 10^{-6}	0.000136	0.000053	0.0099	Skin pigment
rs1545397	0.001355	0.000116	1.28 x 10^{-31}	-0.00051	0.000076	2.30 x 10^{-11}	Skin pigment
rs1800414	0.004959	0.000365	4.57 x 10^{-42}	-0.00233	0.00024	2.94 x 10^{-22}	Skin pigment
rs1800407	0.000661	0.000117	1.47 x 10^{-8}	0.000463	0.000077	1.53 x 10^{-9}	Both
rs12441727	0.002183	0.000087	2.97 x 10^{-137}	-0.00056	0.000058	4.39 x 10^{-22}	Skin pigment
rs1470608	0.002913	0.000076	6.58 x 10^{-320}	-0.00044	0.000050	1.38 x 10^{-18}	Skin pigment
rs1129038	-0.0051	0.000061	<2.23 x 10^{-308}	0.000158	0.000042	0.00019	Skin pigment
rs12913832	-0.00509	0.000061	<2.23 x 10^{-308}	0.00016	0.000042	0.00016	Skin pigment
rs2238289	0.003329	0.000078	<2.23 x 10^{-308}	0.000178	0.000052	0.00057	Skin pigment
rs6497292	0.004373	0.000112	<2.23 x 10^{-308}	-0.000086	0.000074	0.25	Skin pigment
rs1667394	-0.00404	0.000070	<2.23 x 10^{-308}	-0.000022	0.000047	0.65	Skin pigment
rs1426654	0.003598	0.000399	2.05 x 10^{-19}	-0.00317	0.000262	1.46 x 10^{-33}	Skin pigment
rs3114908	0.000754	0.000066	4.51 x 10^{-30}	-0.00015	0.000044	0.00062	Skin pigment
rs3212355	0.004681	0.000927	4.41 x 10^{-7}	-0.00222	0.000609	0.00027	Skin pigment
rs1805006	-0.00194	0.000422	4.36 x 10^{-6}	0.002331	0.000277	4.19 x 10^{-17}	Skin pigment
rs2228479	-0.00135	0.000101	9.24 x 10^{-41}	0.000051	0.000067	0.44	Skin pigment
rs11547464	0.001844	0.000656	0.0049	-0.00039	0.000431	0.36	Skin pigment
rs1805007	-0.00136	0.000115	2.70 x 10^{-32}	0.000958	0.000076	1.28 x 10^{-36}	Both
rs1110400	-0.00147	0.000671	0.029	0.000088	0.000441	0.84	Skin pigment
rs1805008	-0.00127	0.000107	4.13 x 10^{-32}	-0.000075	0.000071	0.29	Skin pigment
rs885479	-0.00096	0.000146	4.45 x 10^{-11}	-0.00062	0.000096	1.38 x 10^{-10}	Skin pigment
rs8051733	-0.00087	0.000065	2.96 x 10^{-41}	0.000276	0.000043	7.87 x 10^{-11}	Skin pigment
rs6059655	0.002929	0.000103	3.44 x 10^{-176}	-0.00147	0.000068	6.76 x 10^{-104}	Both
rs6119471	0.006128	0.000431	7.02 x 10^{-46}	-0.00041	0.000283	0.14	Skin pigment
rs2378249	0.001761	0.000076	8.04 x 10^{-120}	-0.00054	0.000050	1.01 x 10^{-27}	Skin pigment
rs62246017	0.001119	0.000061	4.34 x 10^{-76}	0.000076	0.000040	0.056	cSCC
rs6791479	-0.00126	0.000056	3.89 x 10^{-114}	0.000108	0.000037	0.0031	cSCC
rs35407	-0.00684	0.000145	<2.23 x 10^{-308}	0.000908	0.000097	6.24 x 10^{-21}	Both
rs4455710	0.000956	0.000057	7.34 x 10^{-64}	-0.00037	0.000037	2.66 x 10^{-23}	cSCC
rs117132860	0.003098	0.000226	1.07 x 10^{-42}	0.00028	0.000149	0.060	cSCC
rs10810657	-0.00113	0.000056	4.99 x 10^{-89}	0.000667	0.000037	3.96 x 10^{-72}	Both
rs57994353	-0.00013	0.000073	0.069	-0.000096	0.000048	0.044	cSCC
rs12916300	-0.00455	0.000059	<2.23 x 10^{-308}	0.000077	0.000041	0.058	Both
rs4268748	-0.0013	0.000068	2.71 x 10^{-80}	0.000228	0.000045	3.89 x 10^{-7}	Both

*Each SNP was associated with skin pigment trait, cSCC risk, or both (skin pigment and cSCC) in the current study.

Supplementary Table 7. European ancestry association with cSCC risk at known cSCC- and/or skin pigmentation-associated loci in Hispanic/Latinos.

**Chr**	**Locus**	**T**	**P**
3	*FOXP1*	0.0046	1.00
3	*TPRG1-TP63*	-0.20	0.84
5	*SLC45A2*	-0.14	0.89
6	*IRF4*	0.69	0.49
6	*HLA-DQA1*	-1.01	0.31
7	*AHR*	1.01	0.31
9	*TYRP1*	1.44	0.15
9	*BNC2-CNTLN*	1.51	0.13
9	*SEC16A*	-0.35	0.73
11	*TYR*	0.46	0.64
12	*KITLG*	-0.75	0.45
14	*SLC24A4*	-2.35	0.0186
15	*SLC24A5*	3.76	0.000171
15	*OCA2-HERC2*	0.036	0.97
16	*MC1R-DEF8*	0.68	0.49
20	*RALY-ASIP-PIGU*	0.38	0.71

The comparison of the European ancestry percentages for cSCC cases and controls for each lead SNP was assessed using a non-parametric test statistic for admixture mapping (Montana and Pritchard AJHG 2004).

Supplementary Table 8. Multivariate logistic regression model of cSCC stratified by anatomical site (sun exposed or sun protected) in GERA non-Hispanic whites and Hispanic/Latinos

	**non-Hispanic whites**				**Hispanic/Latinos**			
	**Tumor location sun-exposed**		**Tumor location sun-protected**		**Tumor location sun-exposed**		**Tumor location sun-protected**	
	**β (SE)**	***P***	**β (SE)**	***P***	**β (SE)**	***P***	**β (SE)**	***P***
**Model 1: Ancestry (age, sex, and PCs as covariates)**								
**Age**	0.068 (0.0011)	$<2.23 \times 10^{-308}$	0.039 (0.0043)	5.05×10^{-20}	0.078 (0.0060)	5.68×10^{-38}	0.038 (0.019)	0.041
**Sex**	-0.53 (0.023)	4.10×10^{-118}	-0.58 (0.10)	1.22×10^{-8}	-0.59 (0.14)	2.01×10^{-5}	0.19 (0.52)	0.72
**PC1**	30.62 (1.88)	6.16×10^{-60}	20.37 (7.84)	0.0094	127.68 (9.72)*	2.11×10^{-39}	47.93 (28.64)*	0.094
**PC2**	-25.82 (1.81)	4.83×10^{-46}	-17.07 (7.56)	0.0239	72.29 (21.71)*	0.00087	-5.27 (18.44)*	0.78
**PC3**	-13.09 (1.84)	1.19×10^{-12}	-26.94 (7.98)	0.00074	23.50 (13.36)	0.079	-5.95 (34.72)	0.86
**PC4**	0.54 (1.97)	0.78	1.24 (8.65)	0.89	2.63 (7.73)	0.73	38.04 (25.94)	0.14
**PC5**	3.80 (2.37)	0.11	-4.55 (9.63)	0.64	24.53 (90.7)	0.0068	15.68 (24.84)	0.53
**PC6**	1.64 (2.09)	0.43	-1.61 (9.03)	0.86	-11.23 (8.31)	0.18	20.83 (27.86)	0.45
**PC7**	2.98 (2.09)	0.15	2.68 (9.24)	0.77	-	-	-	-
**PC8**	-2.30 (2.10)	0.27	-8.15 (9.33)	0.38	-	-	-	-
**PC9**	-2.34 (2.09)	0.26	3.49 (9.34)	0.71	-	-	-	-
**PC10**	2.56 (2.12)	0.23	10.15 (9.39)	0.28	-	-	-	-
**ASHK**	0.87 (0.09)	2.08×10^{-23}	0.19 (0.39)	0.62	-	-	-	-
**Model 2: Model 1 and HIrisPlex-S skin pigmentation prediction**								
**Age**	0.069 (0.001)	$<2.23 \times 10^{-308}$	0.04 (0.004)	2.36×10^{-20}	0.080 (0.006)	4.10×10^{-38}	0.039 (0.019)	0.04
**Sex**	-0.53 (0.02)	7.86×10^{-118}	-0.58 (0.10)	1.06×10^{-8}	-0.59 (0.14)	2.82×10^{-5}	0.18 (0.52)	0.73
**PC1**	17.02 (1.95)	2.93×10^{-18}	9.83 (8.20)	0.23	70.96 (12.16)	5.41×10^{-9}	37.84 (37.87)	0.32
**PC2**	-19.97 (1.84)	1.71×10^{-27}	-11.85 (7.64)	0.12	56.36 (21.71)*	0.01	-7.16 (19.31)*	0.71
**PC3**	-9.62 (1.86)	2.17×10^{-7}	-23.72 (7.98)	0.0030	20.45 (12.89)	0.11	-6.79 (34.51)	0.84
**PC4**	-0.05 (1.98)	0.98	0.59 (8.64)	0.95	-7.68 (7.77)	0.32	41.12 (26.76)	0.12
**PC5**	3.89 (2.38)	0.10	-4.16 (9.63)	0.67	22.79 (9.07)	0.01	15.36 (24.99)	0.54
**PC6**	1.45 (2.12)	0.49	-1.99 (9.11)	0.83	-10.14 (8.31)	0.22	21.78 (27.74)	0.43
**PC7**	2.36 (2.09)	0.26	2.07 (9.18)	0.82	-	-	-	-
**PC8**	-1.99 (2.11)	0.35	-7.84 (9.33)	0.40	-	-	-	-
**PC9**	-2.61 (2.12)	0.22	3.22 (9.38)	0.73	-	-	-	-
**PC10**	0.16 (2.15)	0.94	8.37 (9.45)	0.38	-	-	-	-
**ASHK**	0.56 (0.09)	2.23×10^{-10}	-0.04 (0.40)	0.91	-	-	-	-
**Intermediate skin**	-1.32 (0.05)	4.46×10^{-136}	-1.15 (0.23)	5.11×10^{-7}	-2.35 (0.33)	1.80×10^{12}	1.10 (1.86)	0.56
**Dark skin**	-2.21 (0.18)	2.11×10^{-36}	-1.37 (0.67)	0.04	-3.30 (0.42)	3.32×10^{-15}	0.21 (1.89)	0.91

Note: The first two principal components (PC1 and PC2), which represent geographic origin, are highlighted in yellow. *$P < 0.05$ for a test of equivalence of the beta coefficients between sun protected tumor locations and sun exposed tumor locations, within ethnicity group and within principal component (i.e., in model 1, there is a significant difference in the beta coefficients of PC1 for sun exposed and sun protected tumor locations in Hispanic/Latinos. The same is true of the beta coefficients of PC2 in Hispanic/Latinos in both models 1 and 2).

Supplementary Table 9. Characteristics of the non-cutaneous SCC cases and controls from GERA cohort

	**ncSCC Cases**	**ncSCC Controls**
Age at specimen (years) Mean ± SD	60.0 ± 14.9	60.7 ± 13.8
N (proportion that are cases)	1,065 (1.39)	75,601
Sex		
Female	774 (1.6%)	46,672
Male	291 (1.0%)	28,929
Ethnicity		
Non-Hispanic White	810 (1.4%)	57,587
Hispanic/Latino	106 (1.3%)	7,784
East Asian	90 (1.2%)	7,151
African American	59 (1.9%)	3,079
Prior AK		
Yes	156 (1.4%)	11,165
No	909 (1.4%)	64,436

Supplementary Table 10. Multivariate logistic regression models of non-cutaneous SCC in GERA non-Hispanic whites and Hispanic/Latinos

	**non-Hispanic whites**		**Hispanic/Latinos**	
**Variable**	**β (SE)**	***P***	**β (SE)**	***P***
**Model 1: Ancestry (age, sex, and PCs as covariates)**				
Age	0.00033 (0.0026)	0.90	-0.012 (0.0066)	0.082
Sex	0.44 (0.079)	3.25×10^{-8}	0.75 (0.24)	0.0018
PC1	-0.16 (4.73)	0.97	13.29 (11.49)	0.25
PC2	1.00 (5.14)	0.85	1.71 (9.02)	0.85
PC3	-11.37 (6.11)	0.063	6.46 (7.85)	0.41
PC4	-7.39 (6.29)	0.24	9.12 (10.97)	0.41
PC5	1.77 (7.32)	0.81	0.60 (9.29)	0.95
PC6	-5.04 (6.20)	0.42	-10.89 (9.39)	0.25
PC7	7.12 (6.79)	0.29	-	-
PC8	2.98 (6.70)	0.66	-	-
PC9	13.87 (6.53)	0.034	-	-
PC10	-4.49 (6.48)	0.49	-	-
ASHK	-0.19 (0.24)	0.43	-	-
**Model 2: Model 1 and HIrisPlex-S skin pigmentation prediction**				
Age	0.0003 (0.003)	0.90	-0.012 (0.007)	0.08
Sex	0.44 (0.08)	3.22×10^{-8}	0.75 (0.24)	0.002
PC1	-0.38 (5.03)	0.94	15.82 (15.33)	0.30
PC2	1.59 (5.18)	0.76	2.28 (9.31)	0.81
PC3	-10.69 (6.12)	0.08	6.66 (7.88)	0.40
PC4	-7.50 (6.29)	0.23	8.65 (11.28)	0.44
PC5	2.17 (7.34)	0.77	0.74 (9.30)	0.94
PC6	-5.63 (6.21)	0.36	-11.15 (9.41)	0.24
PC7	6.90 (6.75)	0.31	-	-
PC8	3.01 (6.68)	0.65	-	-
PC9	13.72 (6.52)	0.04	-	-
PC10	-4.48 (6.48)	0.49	-	-
ASHK	-0.19 (0.24)	0.44	-	-
Intermediate skin	-0.24 (0.18)	0.16	-0.20 (0.75)	0.79
Dark skin	0.17 (0.34)	0.62	-0.03 (0.72)	0.97

Abbreviations: PC, principal component; β, beta; SE, standard error; *P*, *P*-value; NHW, non-Hispanic whites; H/L, Hispanic/Latinos.

Note: The first two principal components (PC1 and PC2), which represent geographic origin, are highlighted in yellow.

Supplementary Table 11. Multivariate logistic regression models of cSCC in GERA Hispanic/Latinos using a weighted or unweighted genetic risk score (GRS)

	**Model using weighted GRS**		**Model using unweighted GRS**	
	**β (SE)**	***P***	**β (SE)**	***P***
**Age**	0.078 (0.006)	$7.08x10^{-42}$	0.078 (0.0058)	$7.89x10^{-42}$
**Sex**	-0.54 (0.13)	$3.33x10^{-5}$	-0.54 (0.13)	$3.47x10^{-5}$
**PC1**	56.54 (11.49)	$8.54x10^{-7}$	57.36 (11.46)	$5.64x10^{-7}$
**PC2**	28.44 (15.63)	0.069	28.33 (15.65)	0.070
**PC3**	11.38 (11.96)	0.34	11.18 (11.95)	0.35
**PC4**	1.90 (6.68)	0.78	1.95 (6.68)	0.77
**PC5**	20.91 (8.31)	0.01	21.00 (8.31)	0.01
**PC6**	-9.76 (7.63)	0.20	-9.57 (7.64)	0.21
**Intermediate skin**	-1.31 (0.37)	$4.54x10^{-4}$	-1.44 (0.36)	$8.19x10^{-5}$
**Dark skin**	-2.14 (0.46)	$2.93x10^{-6}$	-2.30 (0.44)	$1.95x10^{-7}$
**GRS**	0.13 (0.03)	$1.26x10^{-5}$	0.15 (0.04)	$3.08x10^{-5}$

Ancestry Contour Figures Using Viridis Colors

Figure 1: Ancestry contour figure showing cSCC prevalence by genetic ancestry in GERA. a. Hispanic/Latinos; b. non-Hispanic whites. cSCC prevalence is indicated on a color scale, with bluish colors indicating higher prevalence. Axes reflect the first two principal components of ancestry. Nationality subgroup labels were derived from the Human Genome Diversity Project.

a.

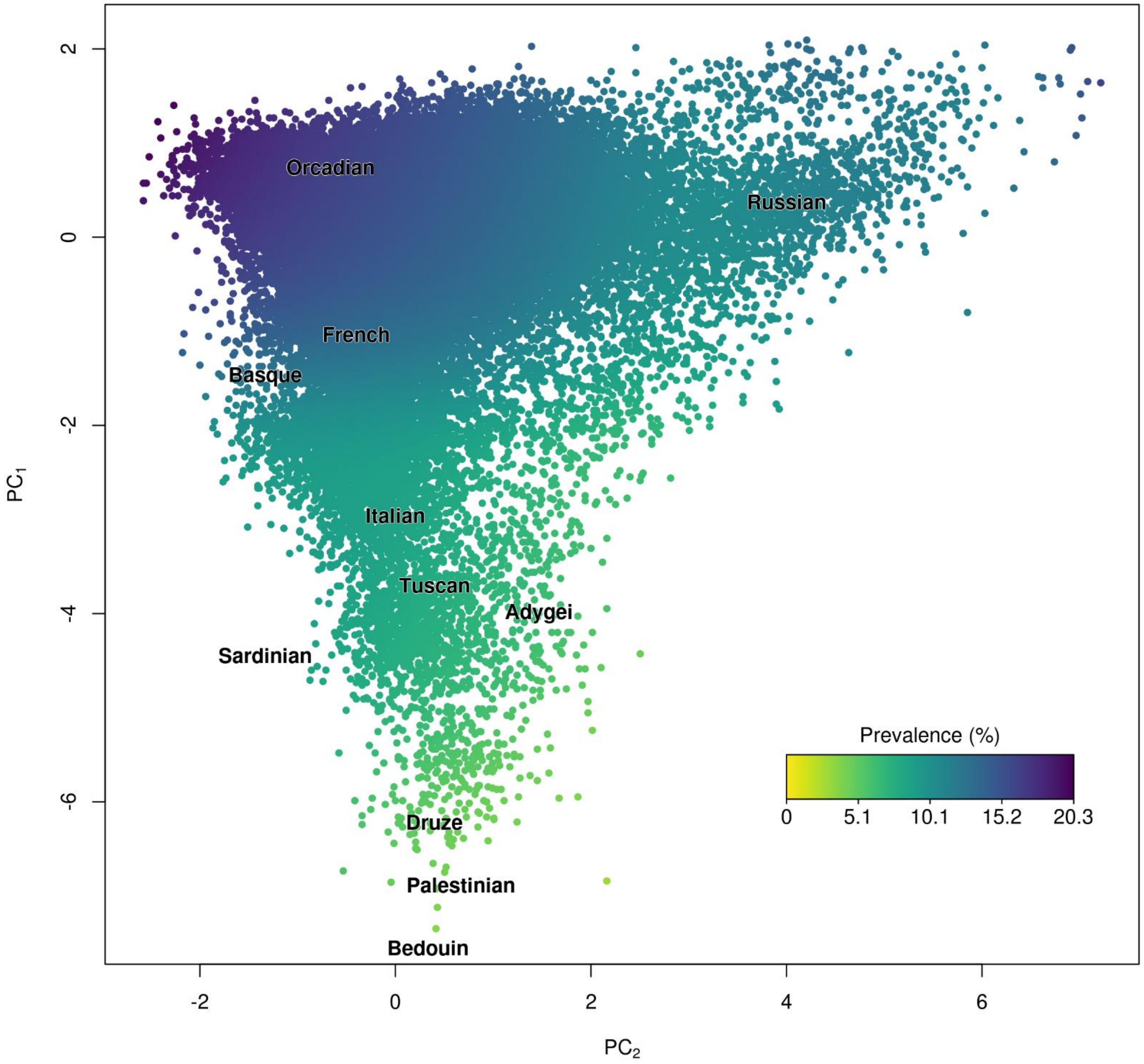

b.

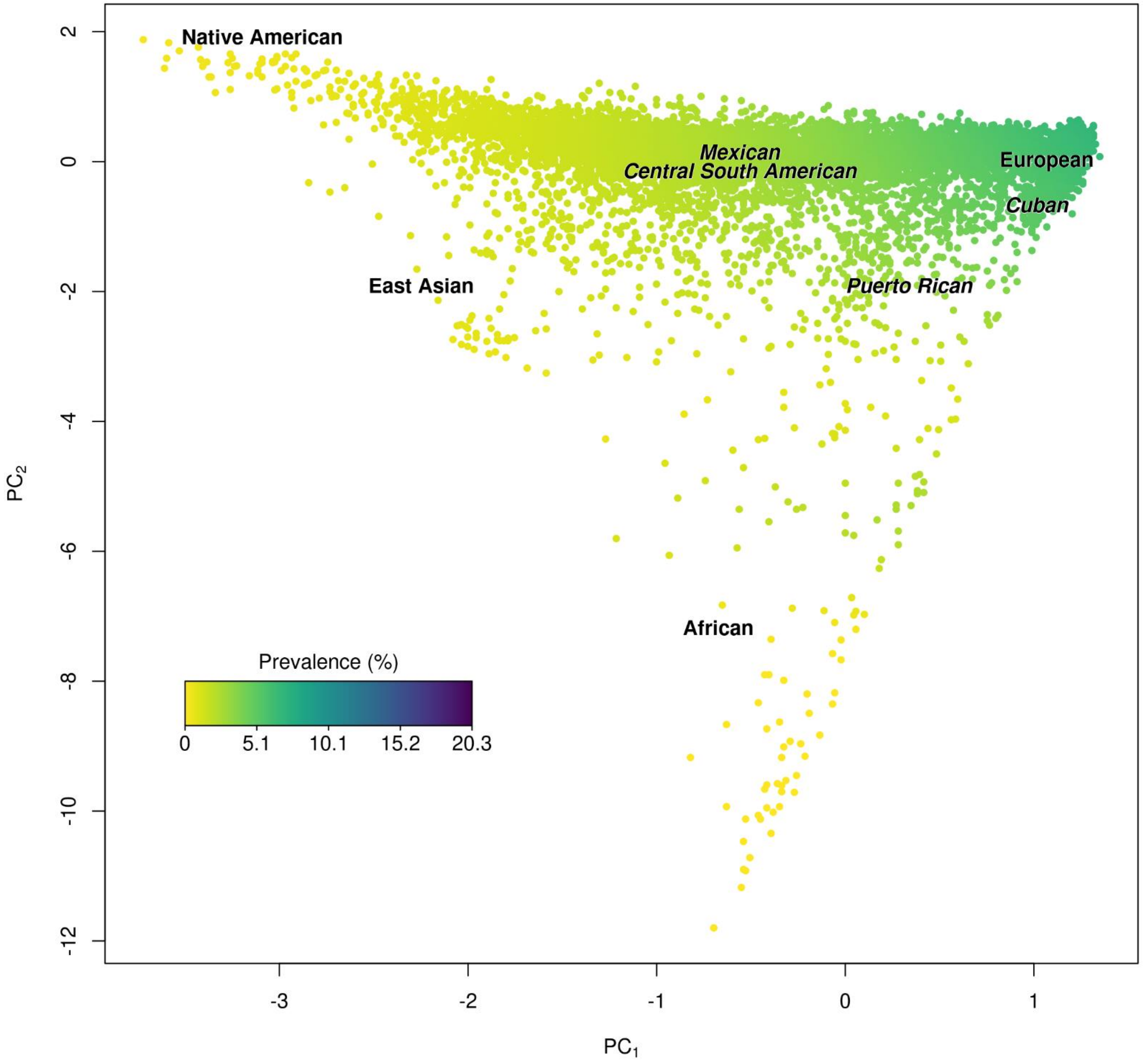

Native American
Mexican
Central South American
European
Cuban
East Asian
Puerto Rican
African
Prevalence (%)
0
5.1
10.1
15.2
20.3
2
0
-2
-4
-6
-8
-10
-12
-3
-2
-1
0
1
PC_1
PC_2

Supplementary Figure 1: Ancestry contour figures showing the probability of each of the skin pigmentation traits versus the first two principal components of genetic ancestry in GERA non-Hispanic whites. a. very pale or pale skin color; b. intermediate skin color; c. dark or dark to black skin color. The probability of each type of skin pigmentation is indicated on a color scale, with bluish colors representing high probabilities of that skin type. Axes reflect the first two principal components of ancestry. Nationality subgroup labels were derived from the Human Genome Diversity Project populations.

a.

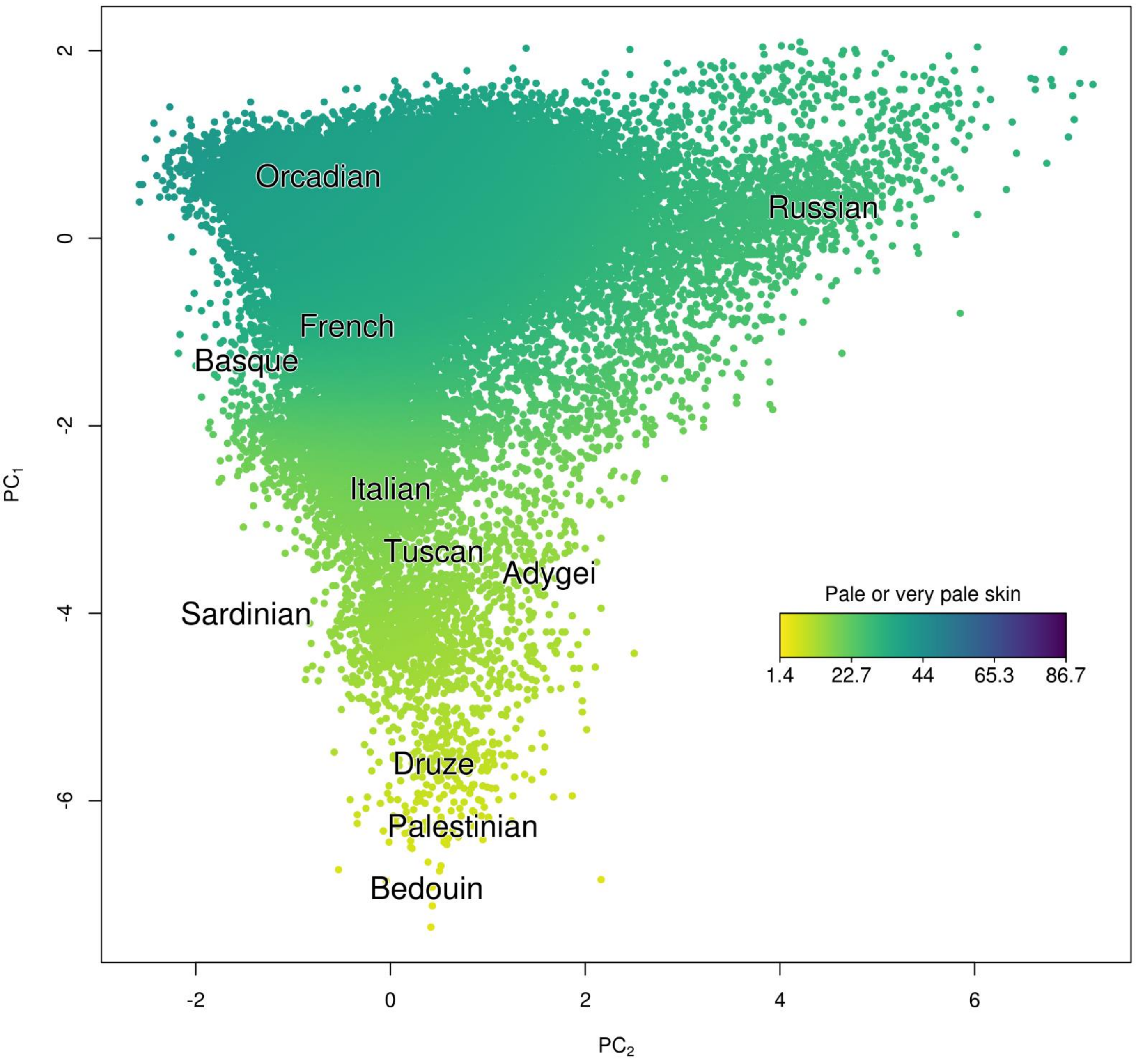

b.

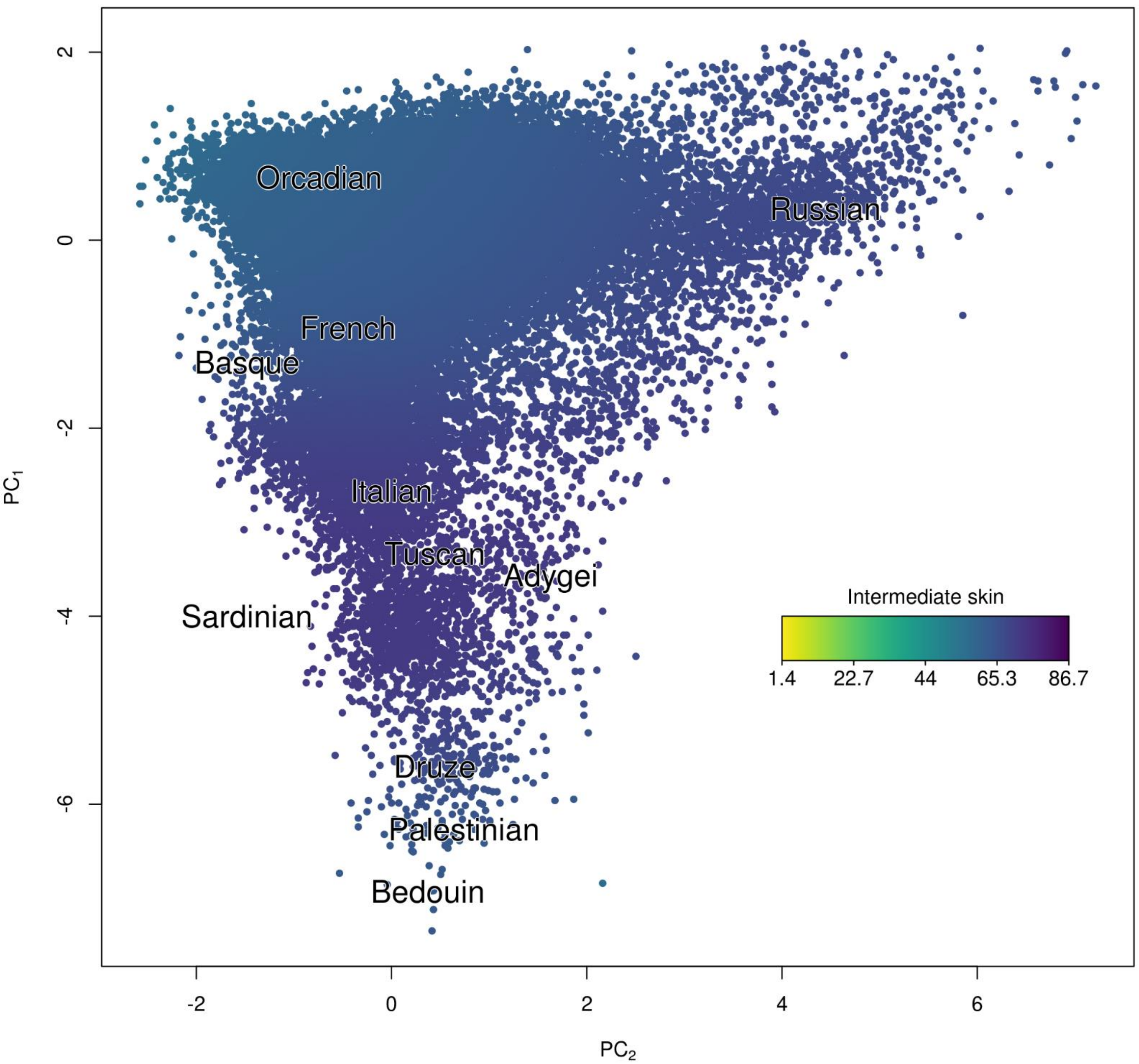

Orcadian
Russian
French
Basque
Italian
Tuscan
Adygei
Sardinian
Druze
Palestinian
Bedouin
Intermediate skin
1.4
22.7
44
65.3
86.7
PC1
PC2
2
0
-2
-4
-6
-2
0
2
4
6

c.

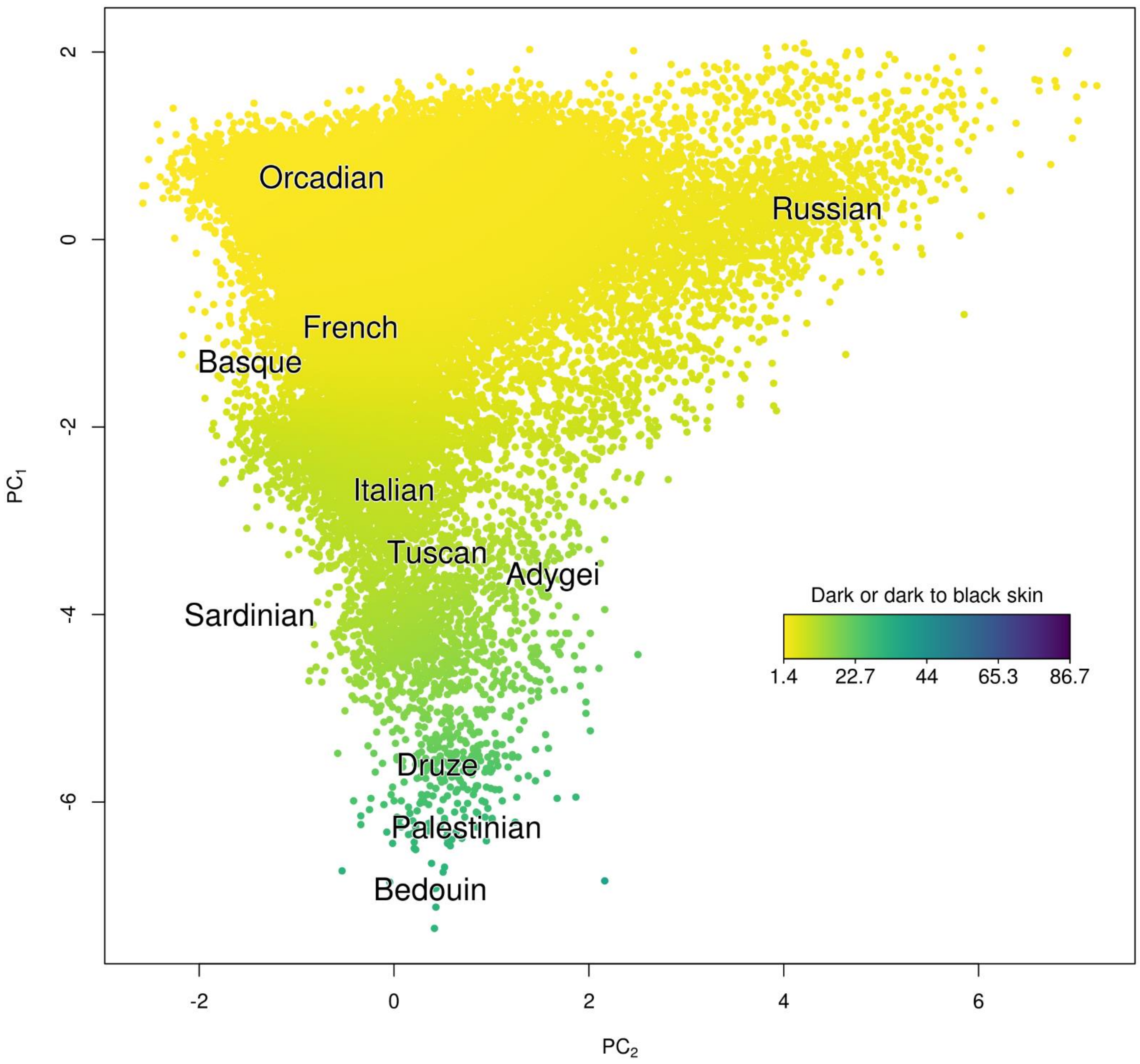

Orcadian
Russian
French
Basque
Italian
Tuscan
Adygei
Sardinian
Druze
Palestinian
Bedouin
Dark or dark to black skin
1.4
22.7
44
65.3
86.7
2
0
-2
-4
-6
-2
0
2
4
6
PC1
PC2

Supplementary Figure 2: Ancestry contour figures showing the probability of each of the skin pigmentation traits versus the first two principal components of genetic ancestry in GERA Hispanic/Latinos. a. very pale or pale skin color; b. intermediate skin color; c. dark or dark to black skin color. The probability of each type of skin pigmentation is indicated on a color scale, with bluish colors representing high probabilities of that skin type. Axes reflect the first two principal components of ancestry. Nationality subgroup labels were derived from the Human Genome Diversity Project populations.

a.

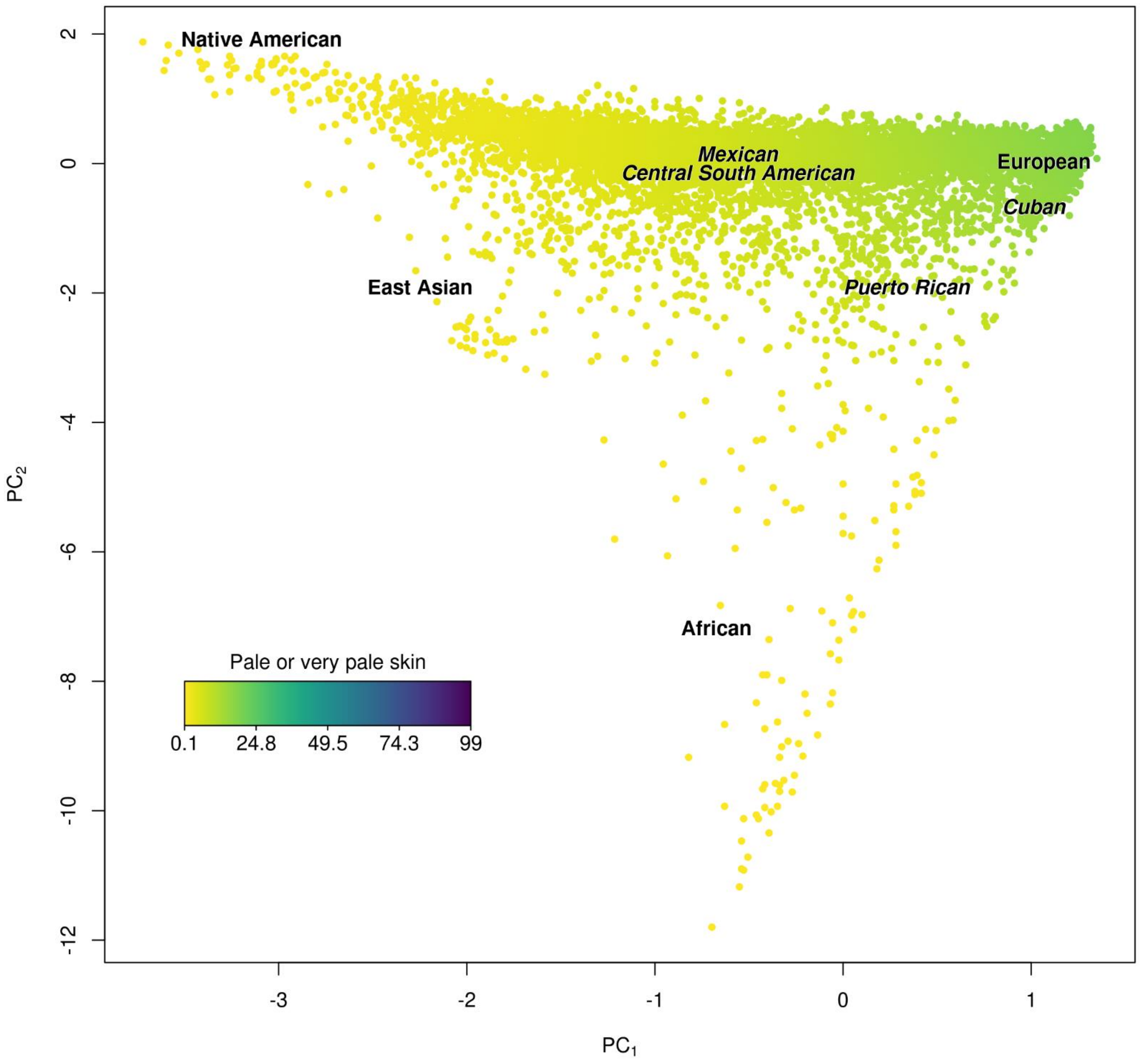

b.

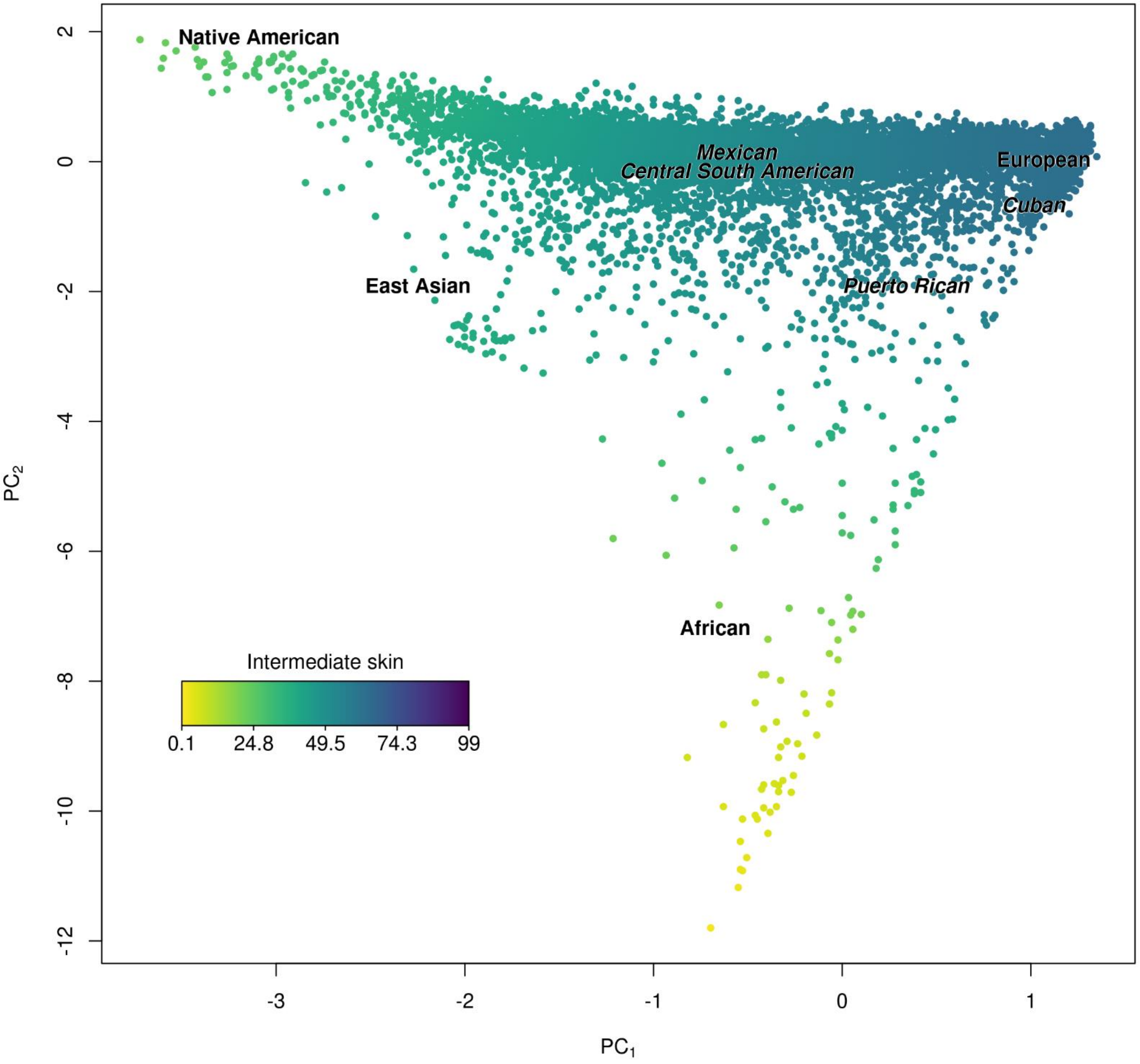

Native American
Mexican
Central South American
European
Cuban
East Asian
Puerto Rican
African
Intermediate skin
0.1
24.8
49.5
74.3
99
2
0
-2
-4
-6
-8
-10
-12
-3
-2
-1
0
1
PC_1
PC_2

c.

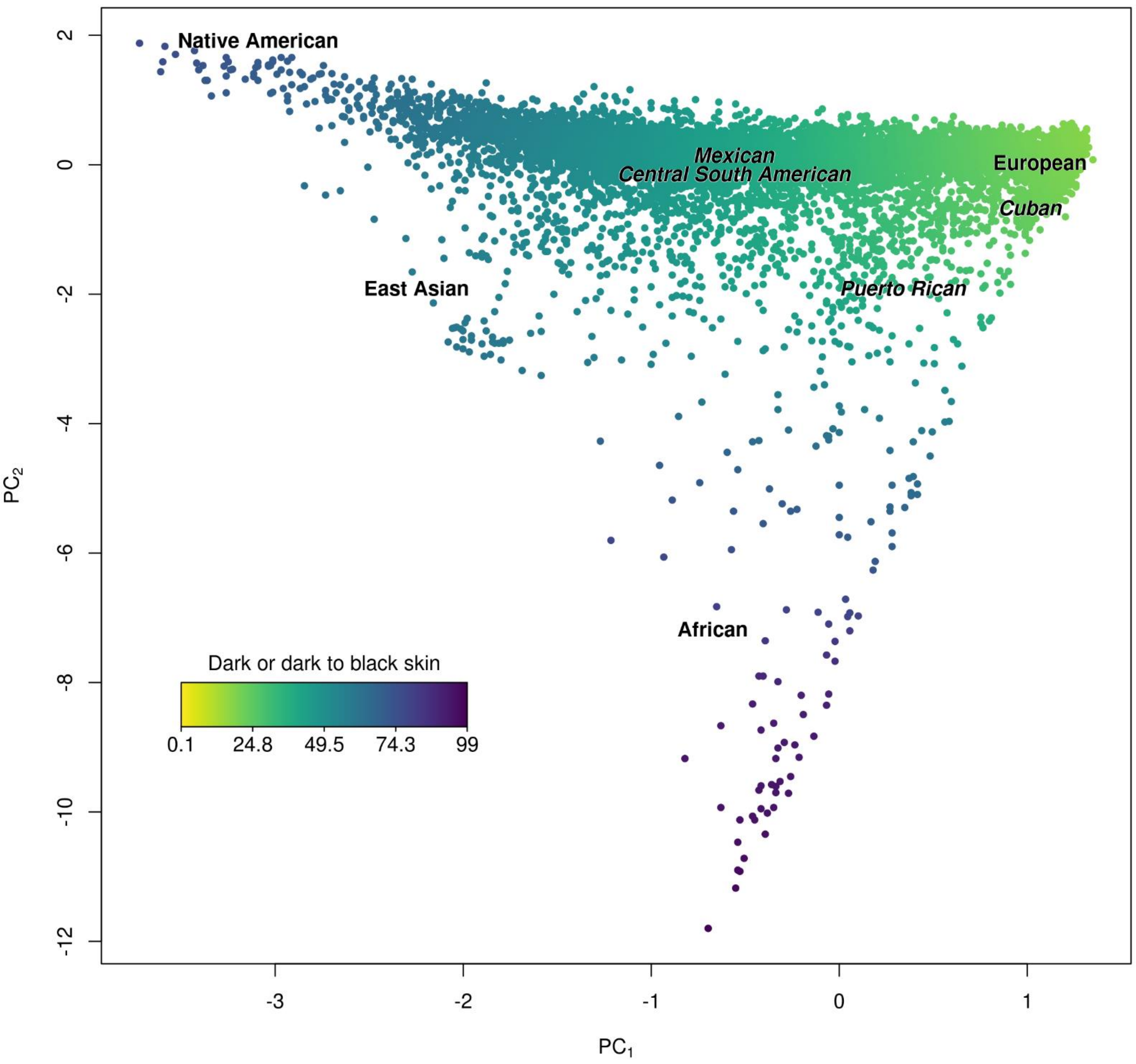

Native American
Mexican
Central South American
European
Cuban
East Asian
Puerto Rican
African
Dark or dark to black skin
0.1
24.8
49.5
74.3
99
2
0
-2
-4
-6
-8
-10
-12
-3
-2
-1
0
1
PC$_2$
PC$_1$

Supplementary Figure 3: Ancestry contour figure showing cSCC prevalence by genetic ancestry, stratified by tumor location (sun exposed or sun protected). a. cSCC risk at sun protected sites within GERA non-Hispanic whites; b. cSCC risk at sun exposed sites within GERA non-Hispanic whites; c. cSCC risk at sun protected sites within GERA Hispanic/Latinos; d. cSCC risk at sun exposed sites within GERA Hispanic/Latinos. cSCC prevalence is indicated on a color scale, with bluish colors indicating higher prevalence. Axes reflect the first two principal components of ancestry. Nationality subgroup labels were derived from the Human Genome Diversity Project populations.

a.

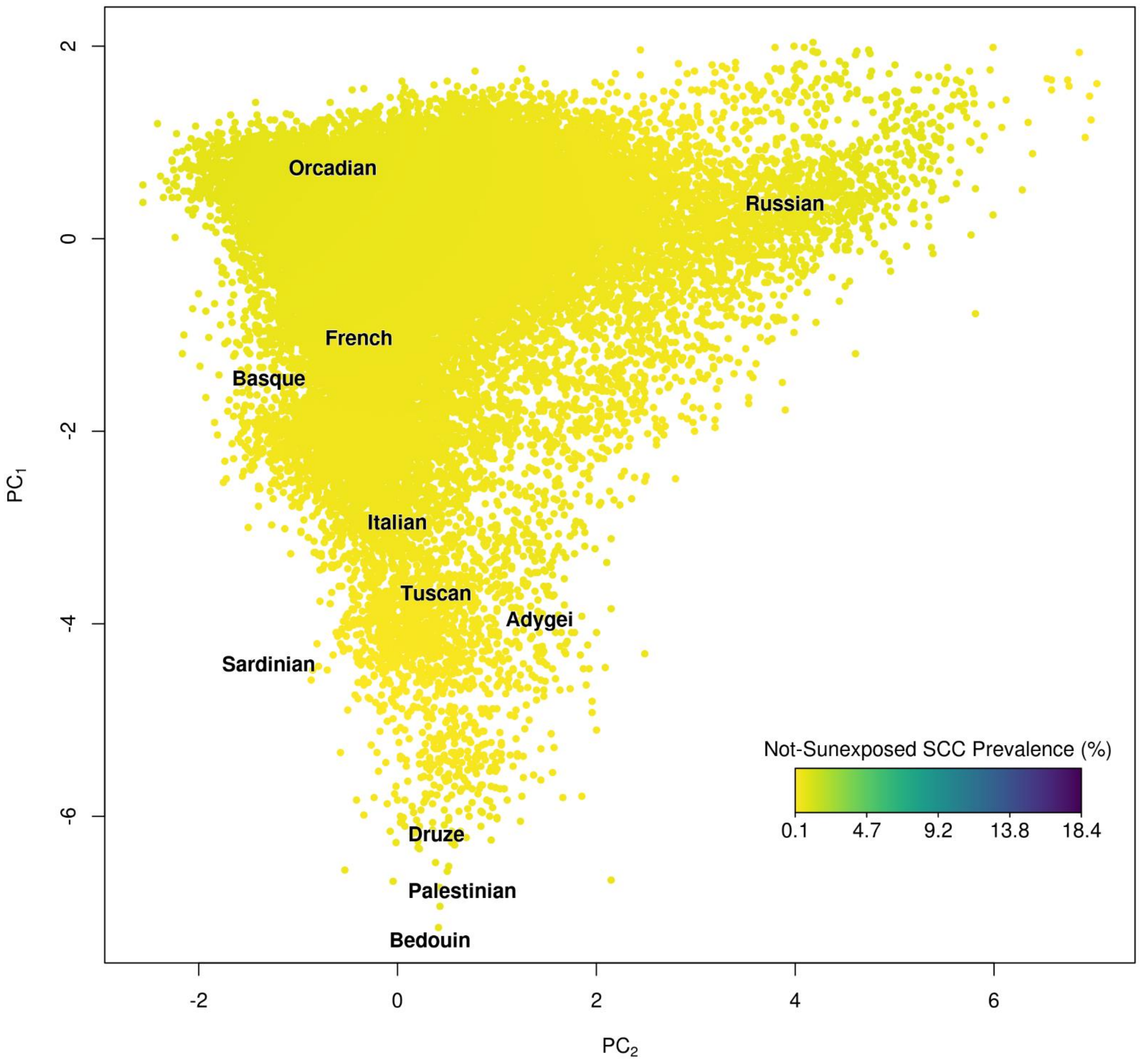

b.

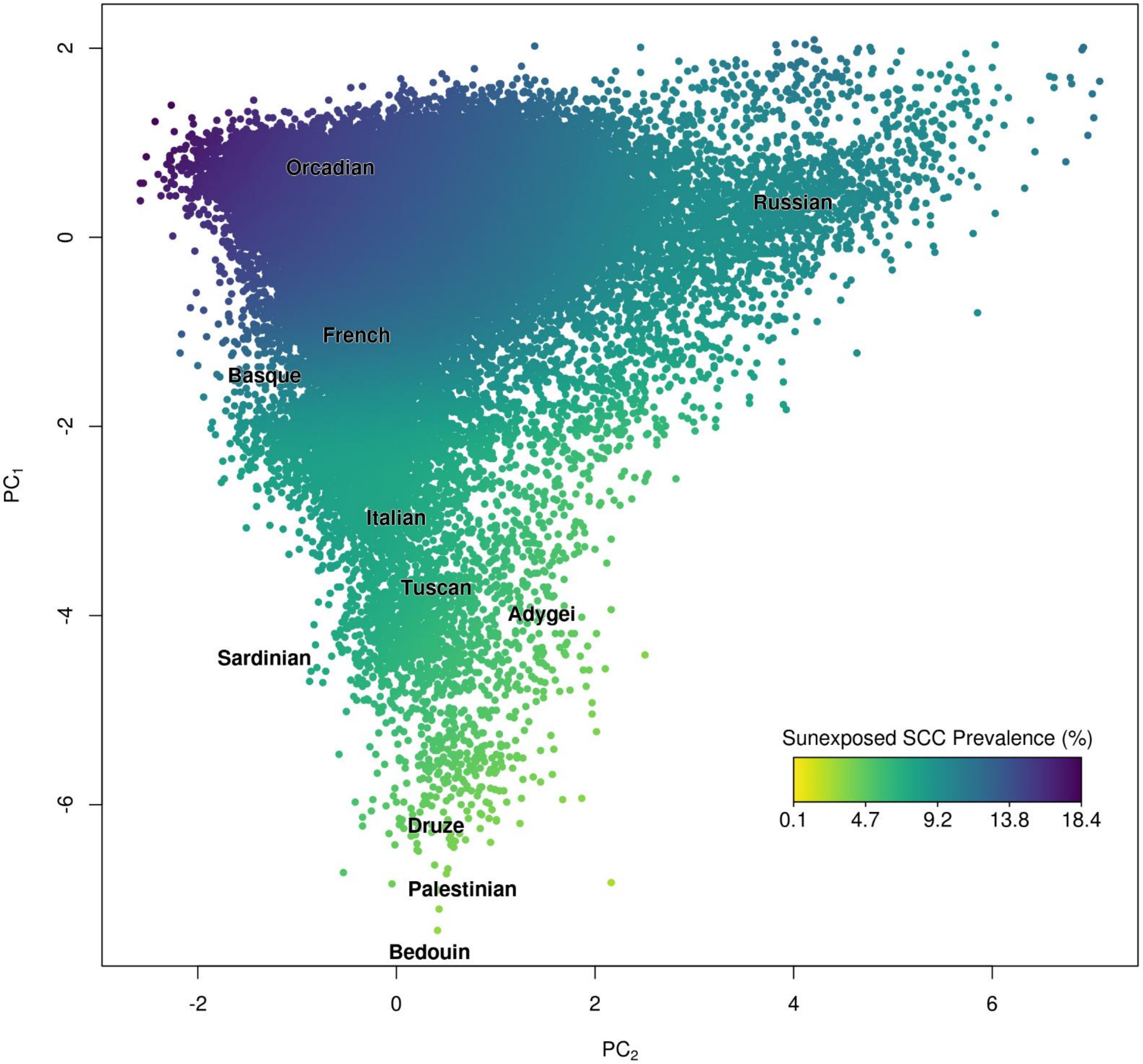

Orcadian
Russian
French
Basque
Italian
Tuscan
Adygei
Sardinian
Druze
Palestinian
Bedouin
Sunexposed SCC Prevalence (%)
0.1
4.7
9.2
13.8
18.4
2
0
-2
-4
-6
-2
0
2
4
6
PC1
PC2

c.

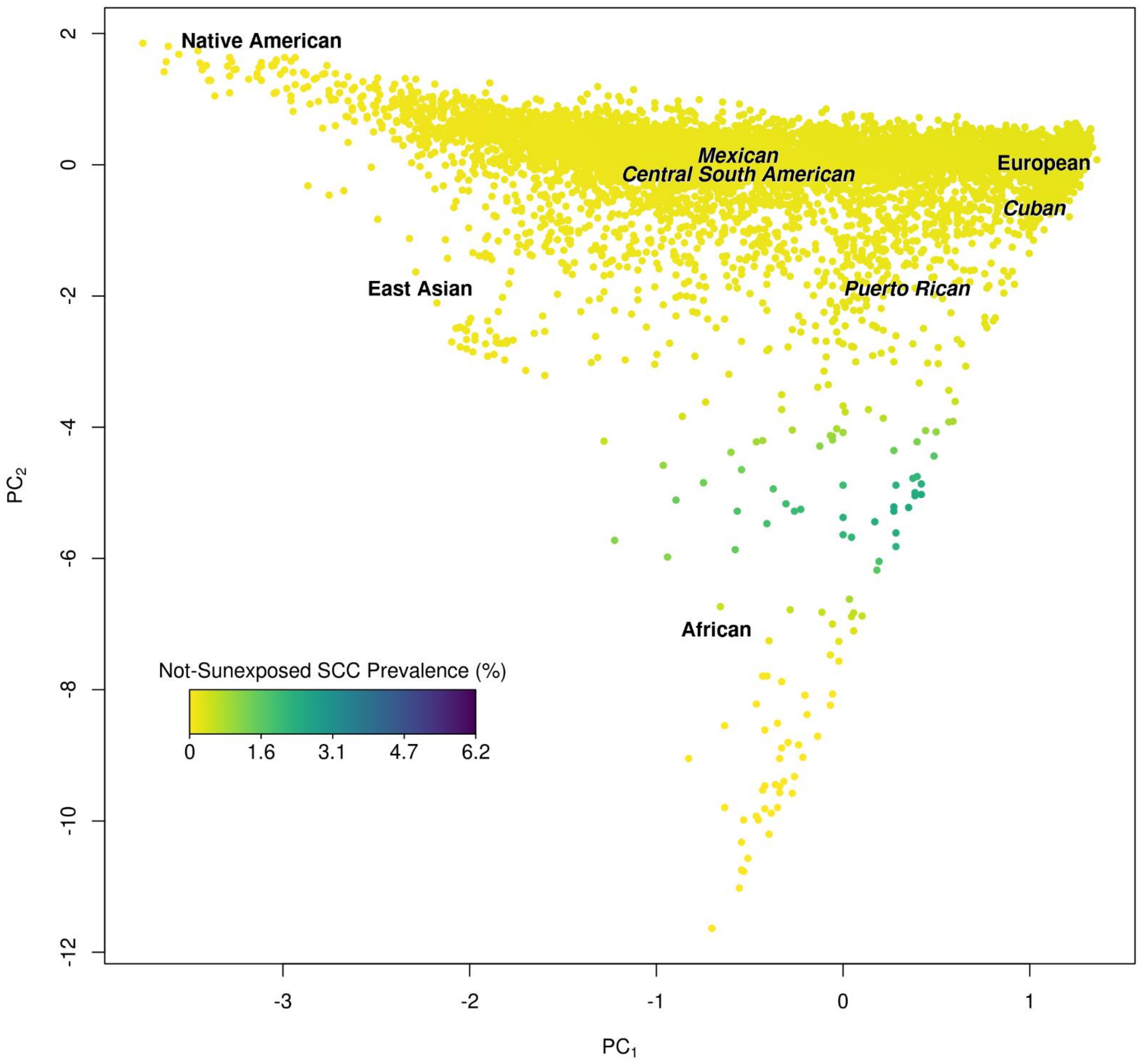

Native American
Mexican
Central South American
European
Cuban
East Asian
Puerto Rican
African
Not-Sunexposed SCC Prevalence (%)
0
1.6
3.1
4.7
6.2
2
0
-2
-4
-6
-8
-10
-12
-3
-2
-1
0
1
PC1
PC2

d.

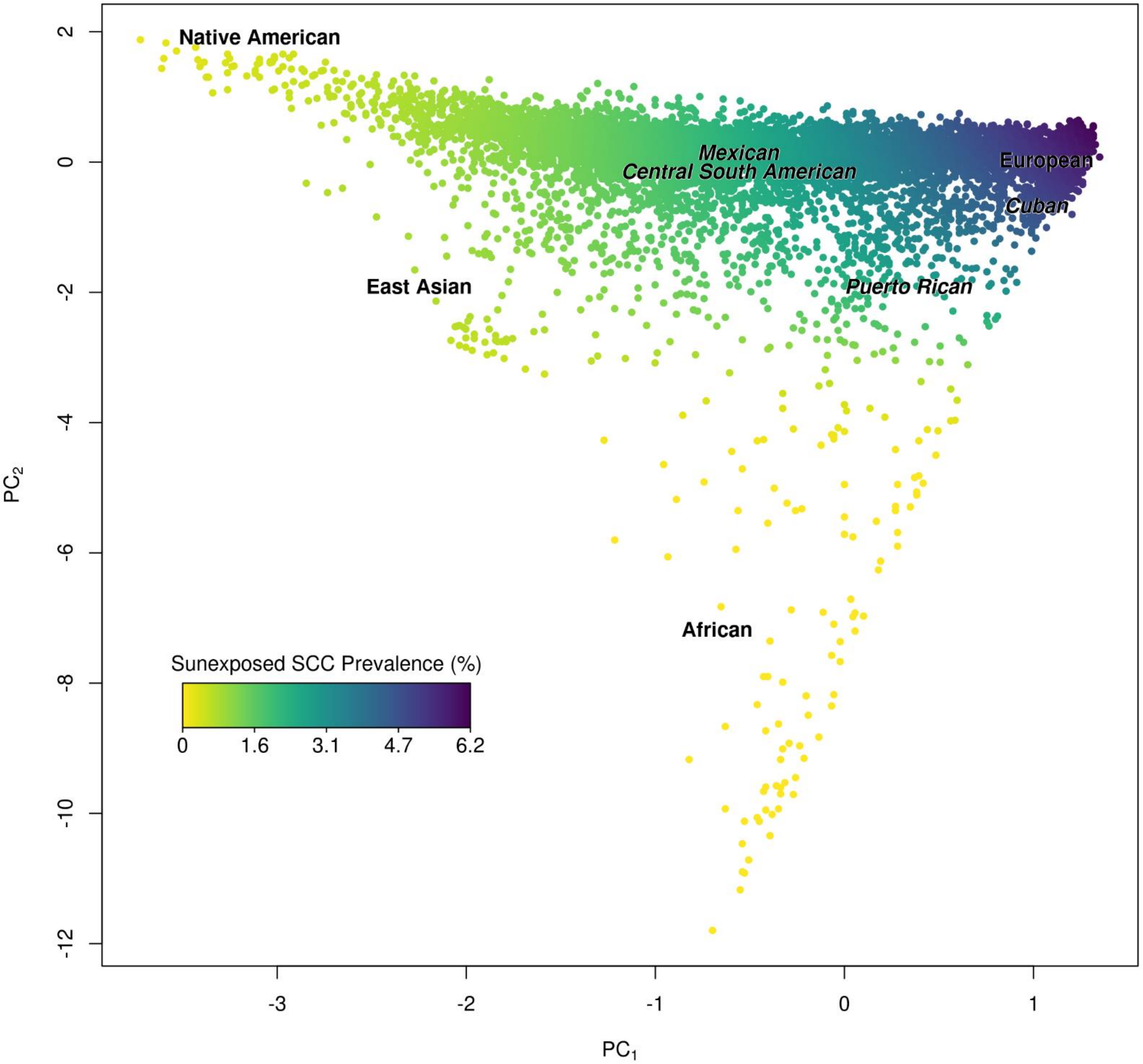
Native American
East Asian
Mexican
Central South American
European
Cuban
Puerto Rican
African
Sunexposed SCC Prevalence (%)
0
1.6
3.1
4.7
6.2
PC2
PC1
2
0
-2
-4
-6
-8
-10
-12
-3
-2
-1
0
1

Supplementary Figure 4: Ancestry contour figure showing non-cutaneous SCC prevalence by genetic ancestry in GERA. a. non-Hispanic whites; b. Hispanic/Latinos. ncSCC prevalence is indicated on a color scale, with warmer colors indicating higher prevalence. Axes reflect the first two principal components of ancestry. Nationality subgroup labels were derived from the Human Genome Diversity Project populations.

a.

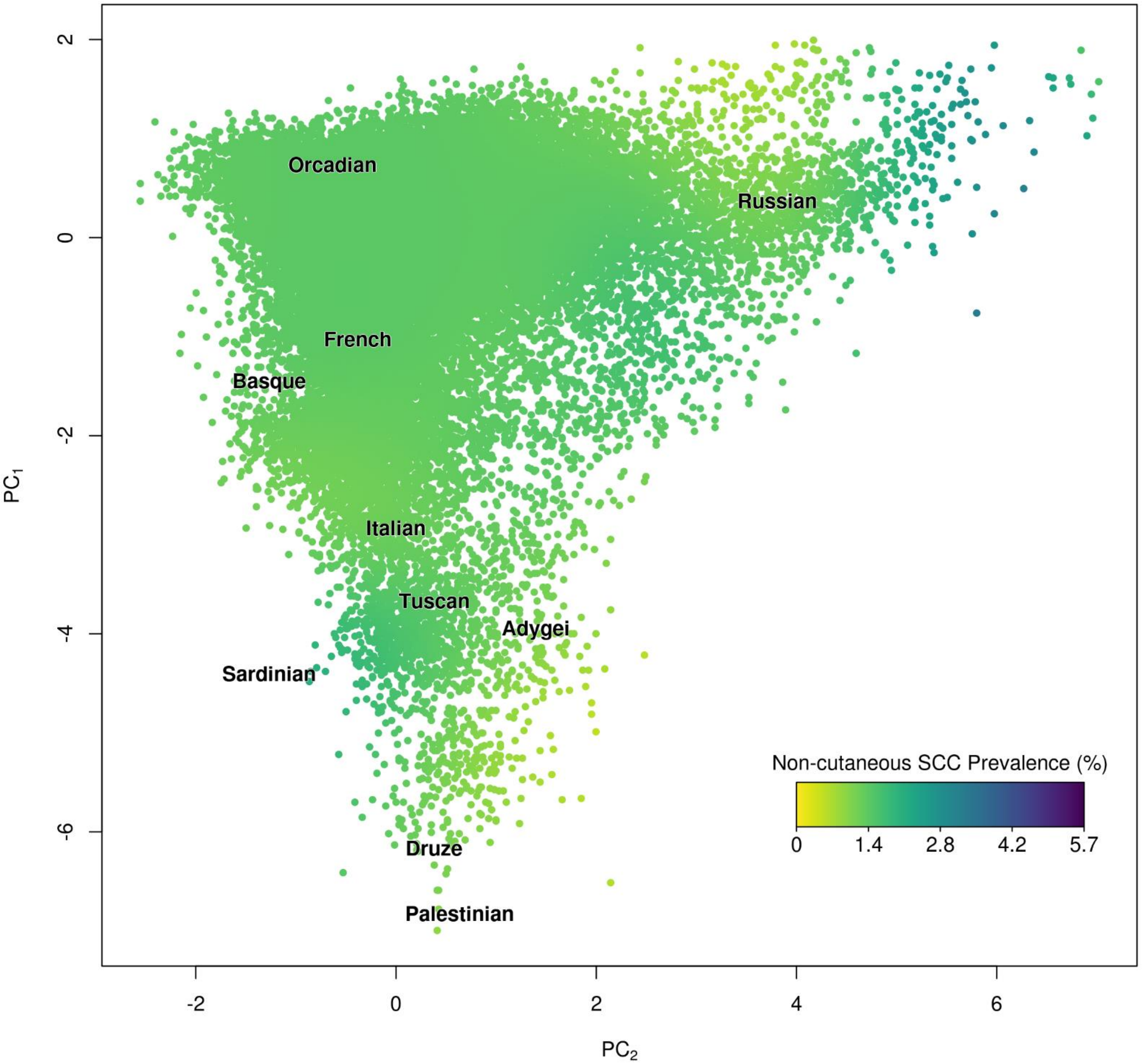

b.

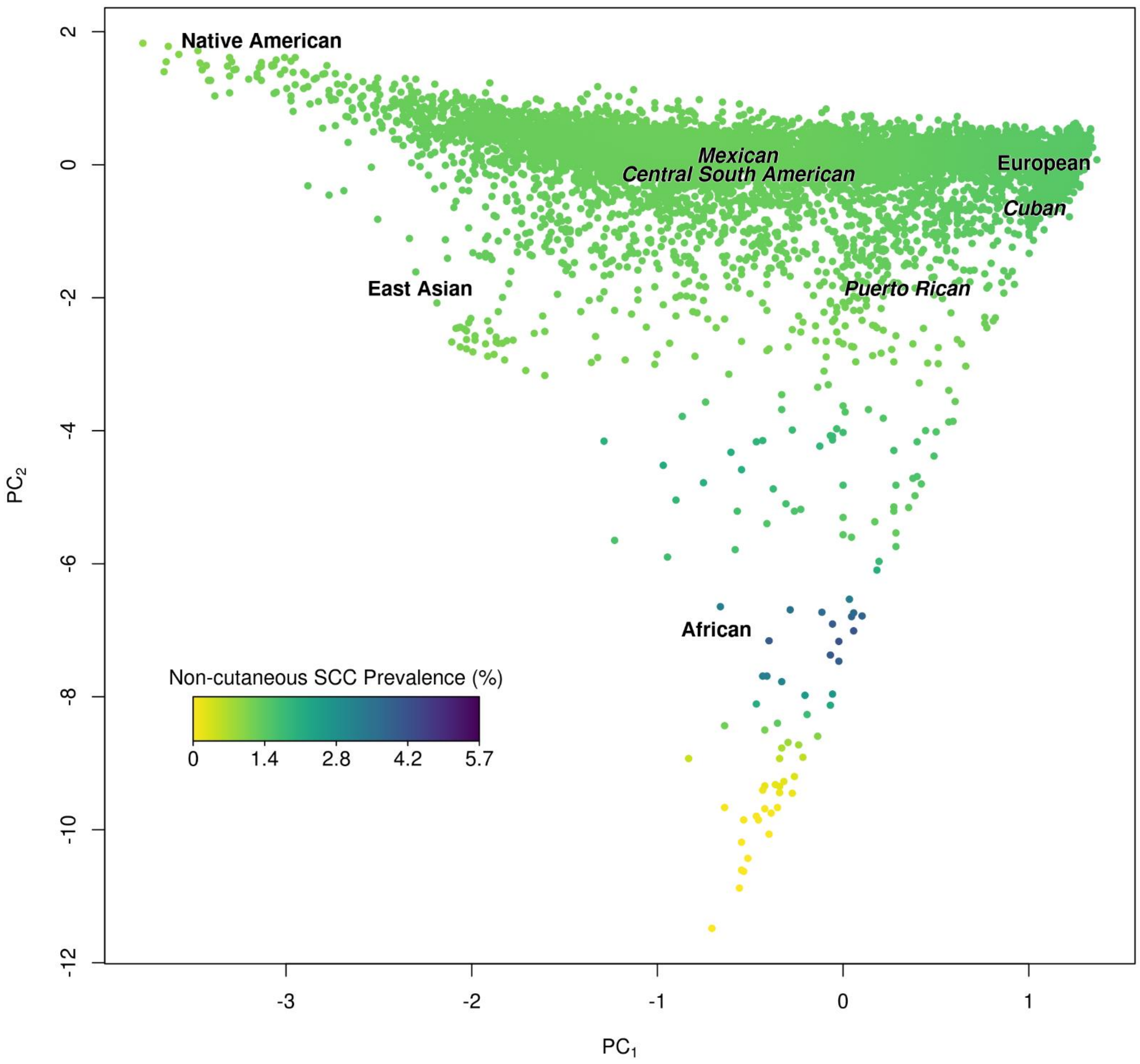

Native American
Mexican
Central South American
European
Cuban
East Asian
Puerto Rican
African
Non-cutaneous SCC Prevalence (%)
0
1.4
2.8
4.2
5.7
PC1
PC2
2
0
-2
-4
-6
-8
-10
-12
-3
-2
-1
0
1
