# Supplementary material for: Genetic ancestry, skin pigmentation, and the risk of cutaneous squamous cell carcinoma in Hispanic/Latino and non-Hispanic white populations
Source: Commun Biol. 2020 Dec 14;3:765. doi: 10.1038/s42003-020-01461-8 (PMC7736583; doi:10.1038/s42003-020-01461-8)
Supplement: Supplementary file 2 — Description of Additional Supplementary Files [file 42003_2020_1461_MOESM2_ESM.pdf]

Description of Additional Supplementary Files

File Name: Supplementary Data 1-8
Description:

- Supplementary Data 1: SNPs associated with human skin pigmentation variation in the HIrisPlexS system and associations with cSCC risk in GERA
- Supplementary Data 2: cSCC-associated SNPs in previous cSCC GWAS studies and replication of associations in GERA
- Supplementary Data 3: cSCC genetic variants and associations with HIrisPlex-S skin pigment probabilities
- Supplementary Data 4: Skin pigmentation genetic variants and associations with cSCC risk after adjusting for 14 cSCC-associated variants (conditional analyses)
- Supplementary Data 5: 95% Credible Set SNPs in GERA Hispanic/Latinos
- Supplementary Data 6: 95% Credible Set SNPs in GERA non-Hispanic whites
- Supplementary Data 7: SNPs associated with human skin pigmentation variation in the HIrisPlexS system and associations with non-cutaneous SCC risk in GERA
- Supplementary Data 8: cSCC-associated SNPs in previous cSCC GWAS studies and associations with non-cutaneous SCC in GERA
