# Supplementary material for: Genetic ancestry, skin pigmentation, and the risk of cutaneous squamous cell carcinoma in Hispanic/Latino and non-Hispanic white populations
Source: Commun Biol. 2020 Dec 14;3:765. doi: 10.1038/s42003-020-01461-8 (PMC7736583; doi:10.1038/s42003-020-01461-8)
Supplement: Supplementary file 4 — Reporting Summary [file 42003_2020_1461_MOESM4_ESM.pdf]

nature research

Corresponding author(s): Hélène Choquet

Last updated by author(s): Oct 5, 2020

Reporting Summary

Nature Research wishes to improve the reproducibility of the work that we publish. This form provides structure for consistency and transparency in reporting. For further information on Nature Research policies, see Authors & Referees and the Editorial Policy Checklist .

Statistics

For all statistical analyses, confirm that the following items are present in the figure legend, table legend, main text, or Methods section.

n/a	Confirmed	
☐	☒	The exact sample size (*n*) for each experimental group/condition, given as a discrete number and unit of measurement
☐	☒	A statement on whether measurements were taken from distinct samples or whether the same sample was measured repeatedly
☐	☒	The statistical test(s) used AND whether they are one- or two-sided<br>*Only common tests should be described solely by name; describe more complex techniques in the Methods section.*
☐	☒	A description of all covariates tested
☐	☒	A description of any assumptions or corrections, such as tests of normality and adjustment for multiple comparisons
☐	☒	A full description of the statistical parameters including central tendency (e.g. means) or other basic estimates (e.g. regression coefficient) AND variation (e.g. standard deviation) or associated estimates of uncertainty (e.g. confidence intervals)
☐	☒	For null hypothesis testing, the test statistic (e.g. *F*, *t*, *r*) with confidence intervals, effect sizes, degrees of freedom and *P* value noted<br>*Give P values as exact values whenever suitable.*
☒	☐	For Bayesian analysis, information on the choice of priors and Markov chain Monte Carlo settings
☐	☒	For hierarchical and complex designs, identification of the appropriate level for tests and full reporting of outcomes
☐	☒	Estimates of effect sizes (e.g. Cohen's *d*, Pearson's *r*), indicating how they were calculated

Our web collection on statistics for biologists contains articles on many of the points above.

Software and code

Policy information about availability of computer code

Data collection	Genotype analysis, quality control, phasing, and imputation analysis were performed on samples from the GERA cohort using the following softwares: Genotyping Console™ Software (Affymetrix) to perform genotype calling, quality control (QC) analysis, and sample or SNP filtering prior to downstream analysis. PLINK software v1.90 to perform additional QC analyses. Shape-IT v2.r727195861 to conduct phasing and IMPUTE2 v2.3.05962 to perform imputation. All software programs employed are available for public use and no custom code was employed.
Data analysis	Eigenstrat v4.2 was used to calculate the principal components (PCs) on each of the four GERA ethnic groups. Pa logistic regression model to examine the impact of ancestry on cutaneous squamous cell carcinoma (cSCC) risk using R version 3.4.1 with the following covariates: age, sex, and ancestry PCs. Skin pigment traits were predicted using HIrisPlex-S, a program that utilizes known pigment genetic variants to generate probabilities of different pigment traits.

For manuscripts utilizing custom algorithms or software that are central to the research but not yet described in published literature, software must be made available to editors/reviewers. We strongly encourage code deposition in a community repository (e.g. GitHub). See the Nature Research guidelines for submitting code & software for further information.

Data

Policy information about availability of data

All manuscripts must include a data availability statement. This statement should provide the following information, where applicable:

- Accession codes, unique identifiers, or web links for publicly available datasets
- A list of figures that have associated raw data
- A description of any restrictions on data availability

Genotype data of GERA participants are available from the database of Genotypes and Phenotypes (dbGaP) under accession phs000674.v2.p2. This includes individuals who consented to having their data shared with dbGaP. The complete GERA data are available upon application to the KP Research Bank (https://researchbank.kaiserpermanente.org/).

Field-specific reporting

Please select the one below that is the best fit for your research. If you are not sure, read the appropriate sections before making your selection.

- [x] Life sciences
- [ ] Behavioural & social sciences
- [ ] Ecological, evolutionary & environmental sciences

For a reference copy of the document with all sections, see nature.com/documents/nr-reporting-summary-flat.pdf

Life sciences study design

All studies must disclose on these points even when the disclosure is negative.

Sample size	A total of 97,582 GERA participants were eligible for analysis, consisting of 11,396 cSCC cases and 86,186 controls from four ethnic groups (80.6% non-Hispanic white, 8.7% Hispanic/Latino, 7.5% East Asian, and 3.2% African American).
Data exclusions	GERA participants who had diagnostic codes for rare genetic disorders associated with increased cSCC risk were excluded.
Replication	The initial study findings which support a shared role of genetic ancestry, skin pigmentation, and sun exposure in cSCC susceptibility in the Hispanic/Latino GERA ethnic group, were confirmed in the non-Hispanic white GERA ethnic group.
Randomization	Allocation of participants was not random in this study. Participants were enrolled and then selected based on pathology records (consistent with incident cSCC for the cases: invasive or in-situ, excluding anogenital and mucosal SCCs; or lack of these pathology records for controls).
Blinding	Blinding was not relevant to our study. Participants were selected based on pathology records.

Reporting for specific materials, systems and methods

We require information from authors about some types of materials, experimental systems and methods used in many studies. Here, indicate whether each material, system or method listed is relevant to your study. If you are not sure if a list item applies to your research, read the appropriate section before selecting a response.

Materials & experimental systems

n/a	Involved in the study	
☒	☐	Antibodies
☒	☐	Eukaryotic cell lines
☒	☐	Palaeontology
☒	☐	Animals and other organisms
☐	☒	Human research participants
☐	☒	Clinical data

Methods

n/a	Involved in the study	
☒	☐	ChIP-seq
☒	☐	Flow cytometry
☒	☐	MRI-based neuroimaging

Human research participants

Policy information about studies involving human research participants

Population characteristics	The Genetic Epidemiology Research in Adult Health and Aging (GERA) cohort consists of 110,266 adult men and women, 18 years and older, who are of non-Hispanic white, Hispanic/Latino, Asian or African American ethnicity.
Recruitment	Participants from the GERA cohort are members of the Kaiser Permanente Northern California (KPNC) integrated health care delivery system, and provided self-reported information via the Research Program on Genes, Environment, and Health (RPGEH) survey.
Ethics oversight	The Institutional Review Board of the Kaiser Foundation Research Institute has approved all study procedures.

Note that full information on the approval of the study protocol must also be provided in the manuscript.

Clinical data

Policy information about clinical studies

All manuscripts should comply with the ICMJEguidelines for publication of clinical research and a completedCONSORT checklist must be included with all submissions.

Clinical trial registration	NA
Study protocol	NA

Data collection	GERA participants included in this study had (or did not have any) pathology records that were consistent with incident cSCC
Outcomes	cSCC cases were defined as subjects whose pathology records were consistent with incident cSCC (invasive or in-situ, excluding anogenital and mucosal SCCs). Controls were subjects with no pathology records consistent with cSCC.
